# Systemic immune inflammation mediates the association of serum omega-3 and omega-6 polyunsaturated fatty acids with biological aging: a national population-based study

**DOI:** 10.1007/s40520-025-02964-2

**Published:** 2025-03-08

**Authors:** Fei Shan, Yu Xiong, Pearl Pai, Mingya Liu

**Affiliations:** 1 Department of Cardiology, University of Hong Kong-Shenzhen Hospital, No.1, Haiyuan 1st Road, Futian District, Shenzhen, Guangdong China; 2 Department of Neurology, University of Hong Kong-Shenzhen Hospital, No.1, Haiyuan 1st Road, Futian District, Shenzhen, Guangdong China; 3 Department of Medicine, University of Hong Kong-Shenzhen Hospital, No.1, Haiyuan 1st Road, Futian District, Shenzhen, Guangdong China

**Keywords:** Polyunsaturated fatty acids, Biological aging, Systemic immune inflammation, NHANES

## Abstract

**Objective:**

This study aimed to explore the association between serum omega-3 (n-3) and omega-6 (n-6) polyunsaturated fatty acids (PUFAs) and biological aging, along with the potential mediating role of systemic immune inflammation (SII).

**Methods:**

Data from the National Health and Nutrition Examination Survey (NHANES) 2011–2014 were used for analyses. Accelerated aging in participants was assessed by calculating the difference between phenotypic age (PhenoAge) and chronological age. Weighted multivariate linear regression models and subgroup analysis were used to investigate the correlation between serum n-3 and n-6 PUFAs and accelerated aging, and restricted cubic spline (RCS) model was applied to explore potential nonlinear relationships. We further conducted mediation analyses to assess the role of SII in these relationships. Additionally, weighted quantile sum (WQS) regression and quantile g-computation (QGC) models were conducted to investigate the mixed effects of serum PUFAs and identify the key contributor.

**Results:**

A total of 3376 participants were enrolled in this study. In multivariate linear regression models, eight of the twelve individual serum PUFAs showed a significantly negative association with PhenoAge acceleration, Specifically, per-unit increases in linoleic acid (LA), gamma-linolenic acid (GLA), arachidonic acid (AA), alpha-linolenic acid (ALA), stearidonic acid (SDA), eicosapentaenoic acid (EPA), docosapentaenoic acid (n-3 DPA), and docosahexaenoic acid (DHA) were all associated with reduced PhenoAge acceleration (*P* < 0.05, respectively). Subgroup analysis demonstrated robust consistence results when stratified by age, sex, and race/ethnicity. L-shaped nonlinear relationships were observed between PhenoAge acceleration with total n-6 PUFAs, LA and ALA (all *P* for nonlinear < 0.05). Mediation analyses indicated that SII mediated the relationship between serum PUFAs and reduced PhenoAge acceleration. Mixed-effects analysis using WQS and QGC models revealed that the combined effect of serum PUFAs on reducing PhenoAge acceleration, with DHA showing the strongest significant contribution.

**Conclusions:**

This study demonstrated that higher levels of certain PUFAs were associated with a reduction in PhenoAge acceleration either individually or in combination, with DHA having the most prominent effect in mixed effects. The SII mediated these relationships, suggesting that PUFAs may slow biological aging by reducing inflammation. These findings highlighted the potential role of PUFAs in mitigating accelerated aging and their implications for aging-related health interventions.

**Supplementary Information:**

The online version contains supplementary material available at 10.1007/s40520-025-02964-2.

## Introduction

As most people can expect to live into their 60 s and over, the average life expectancy has been increasing rapidly [1]. Concurrently, as the aging population becomes more pronounced and many diseases exhibit age-dependency, aging has progressively emerged as a critical public health issue [2]. People who get older frequently face an increased risk of developing chronic diseases such as dementia, cardiovascular disease, diabetes and cancers [3]. These age-related health challenges highlight the importance of evaluating aging status and its impact on human health.

Aging is a complex biological process characterized by the time-dependent accumulation of molecular and cellular damage over the time, resulting in a persistent organismal decline [4]. Although chronological age is undoubtedly a significant risk factor for chronic diseases, individuals of the same chronological age often exhibit differential age‐related organ decline and varying susceptibilities to diseases [5]. Biological age is therefore acknowledged as a more informative marker of disease risk and mortality compared to chronological age [6]. PhenoAge is a biological aging metric developed by Levine et al. based on chronological age and clinical biomarkers [7]. To explain the heterogeneity between biological aging and chronological age, “PhenoAge Acceleration” has been proposed as a tool for evaluating overall health status and age-related decline.

PUFAs are fatty acids with two or more methylene-interrupted double bonds in their acyl chains, typically classified as n-3 and n-6 types. Several lines of evidence suggest that PUFAs, due to their anti-inflammatory and antioxidant effects, play a significant role in the maintenance of good physical performance with aging [8]. Previous studies have shown that a diet rich in PUFAs especially n-3 family were associated with a lower risk of age-related diseases such as cardiovascular disease, inflammatory disease and cancer [9]. Serum fatty acids serve as biomarkers for long-term essential fatty acid intake as they fluctuate in accordance with the fatty acid composition stored in adipose tissue [10]. SII is a novel index that reflects the overall inflammatory and immune state of the body. Li et al. found that higher dietary intake of n-3 and n-6 PUFAs was negatively associated with SII levels [11]. Additionally, Wang et al. reported that higher SII scores were positively correlated with PhenoAge acceleration [12]. Based on these findings, we hypothesize that PUFAs may help slow the process of accelerated aging by reducing inflammation.

Despite growing interest in the role of PUFAs in aging, the specific relationship between serum PUFAs and PhenoAge acceleration, along with the potential mediating effects of inflammation, remains underexplored. This study aimed to fill this gap by examining these associations in a representative sample of US adults.

## Methods

### Study population

NHANES is a cross-sectional, nationally representative survey conducted by the Centers for Disease Control and Prevention (CDC) in the United States to assess the health and nutritional status of the US population. Data are collected every 2 years through questionnaires, physical examinations, and biospecimen collection. This study used the data from the 2011–2012 and 2013–2014 cycles. Participants aged 20 years and older were included, with exclusions for those with missing data on serum PUFAs, PhenoAge measurements, and other covariates. Ethical approval for NHANES was obtained from the National Center for Health Statistics (NCHS) Research Ethics Review Board. All participants in NHANES provided written informed consent. Finally, a total of 3376 participants were included in this study (Supplementary Fig. 1).

### Measurements of serum PUFAs

Serum specimens, collected by trained personnel, were shipped on dry ice and then stored at − 70℃ until analysis. Fasting serum fatty acid levels were measured using an electron capture negative-ion mass spectrometry according to the manufacturer’s instructions. The details about laboratory methodology as well as the quality assurance and monitoring can be found in the NHANES dataset, accessible on the CDC’s website (https://wwwn.cdc.gov/Nchs/Nhanes/2011-2012/FAS_G.htm). For analysis, the following 12 PUFAs were selected, including n-6 PUFAs: linoleic acid (LA, 18:2n-6), gamma-Linolenic acid (GLA, 18:3n-6), eicosadienoic acid (EDA, 20:2n-6), homo-gamma-linolenic acid (HGLA, 20:3n-6), arachidonic acid (AA, 20:4n-6), docosatetraenoic acid (DTA, 22:4n-6), docosapentaenoic acid (DPA, 22:5n-6) and n-3 PUFAs: alpha-linolenic acid (ALA, 18:3n-3), stearidonic acid (SDA, 18:4n-3), eicosapentaenoic acid (EPA, 20:5n-3), docosapentaenoic acid (DPA, 22:5n-3), docosahexaenoic acid (DHA, 22:6n-3). Total n-6 and n-3 PUFAs were calculated by summing the individual compositions, and the n-6 to n-3 ratio was determined by dividing total n-6 by total n-3.

### Measurement of biological aging

Biological age was measured by the PhenoAge algorithm, the most validated method based on the clinical laboratory blood chemistries [7]. PhenoAge was recognized for its high predictiveness of morbidity and mortality and its feasibility for implementation within NHANES. Briefly, the PhenoAge algorithm was developed from elastic-net regression of mortality from analysis of NHANES-III data. For this analysis, we selected eight biomarkers (albumin, alkaline phosphatase, creatinine, glycated hemoglobin, white blood cell count, lymphocyte percentage, mean cell volume, and red cell distribution width) and included chronological age as an independent covariate in the model to calculate biological aging. These biomarkers were chosen based on the biomarker set used in the original research, excluding C-reactive protein (CRP) due to its unavailability in NHANES data from 2011 to 2014. To evaluate the effect of CRP on the calculation of PhenoAge, we compared the results of the algorithm using a biomarker set with and without CRP in NHANES from 1999 to 2010 and found a strong correlation between them, with a correlation coefficient of 0.99 (Supplementary Fig. 2). This was consistent with other studies calculating PhenoAge with the biomarker set without CRP [13, 14]. To assess the difference between biological age and chronological age, PhenoAge Acceleration (PhenoAgeAccel) was determined by calculating the residuals from regressing chronological age on biological age using linear regression. Higher PhenoAgeAccel values indicated accelerated biological aging and increased risk of age-related diseases and mortality. Conversely, lower PhenoAgeAccel values suggest slower aging. The ‘BioAge’ R package was used to compute biological age values. Detailed information is available in Supplementary Method 1.

### Measurement of SII

SII was assessed using the SII index, as in previous studies, which is calculated by multiplying platelet count by neutrophil count and dividing by lymphocyte count [12]. The main components were obtained from the complete blood count (CBC) data in the NHANES dataset, with the measurements were conducted using automated hematology analyzers (Coulter DxH 800 analyzer).

### Covariates

The following potential confounding variables were included in our analysis including age, sex, race/ethnicity, education level, poverty-to-income ratio, body mass index, smoking status, diabetes mellitus, hypertension, cardiovascular disease (CVD) and cancer. Age was defined as the participant’s age at the time of screening. Sex was categorized as male or female. Race/ethnicity was classified as non-Hispanic White, non-Hispanic Black, Mexican American, and Others. Education level was grouped into three categories: less than high school, high school or equivalent, and college or above. The poverty-to-income ratio (PIR), an index used to evaluate household socioeconomic status, was classified as ≤ 1.3, 1.3–3.5, and ≥ 3.5. The body mass index (BMI) was calculated by dividing the participant’s weight in kilograms by their height in meters squared and categorized as three: under/normal weight (≤ 24.9 kg/m^2^), overweight (25–29.9 kg/m^2^), and obese (≥ 30 kg/m^2^). Smoking status was defined as current smokers, former smokers, and never smokers. Never smokers were defined as individuals who had smoked fewer than 100 cigarettes in their lifetime. Former smokers were individuals who had smoked more than 100 cigarettes in their lifetime but currently did not smoke at all. Current smokers were individuals who had smoked more than 100 cigarettes in their lifetime and currently smoked either some days or every day. The presence of diabetes mellitus, hypertension, cancer, and CVD (including congestive heart failure, myocardial infarction, coronary heart disease, and stroke) was determined based on the self-reported chronic medical conditions provided by the participants.

### Statistical analysis

All statistical analyses accounted for NHANES survey design factors, including sample weights, stratification, and clustering. Participants were categorized into two groups based on the presence or absence of accelerated aging. Continuous variables were described with means and SDs (for normally distributed data) or medians with interquartile ranges (IQR) (for skewed distributions). Categorical variables were presented as counts with percentages (%). Differences between groups were assessed by one-way ANOVA (for continuous variables with normal distribution), Kruskal–Wallis tests (for continuous variables with non-normal distribution), and chi-square tests (for categorical variables). Weighted multivariate linear regression models were performed to assess the association between serum n-3 and n-6 PUFAs and PhenoAgeAccel. Serum PUFAs was entered both as a categorical variable (with lowest tertile as a reference) and a continuous variable (with *β* per SD increment). Three models were fitted to adjust for potential confounding variables. Model 1 was adjusted for nothing, while Model 2 was adjusted for age, sex, and race/ethnicity and Model 3 was adjusted for variables in model 2, along with educational level, PIR, smoking status, BMI, diabetes mellitus, hypertension, cancer, and CVD. Results were presented as *β* coefficient with 95% confidence interval (*CI*). Subgroup analyses stratified by age, sex and race/ethnicity were conducted to identify potential effect modifiers in the relationship between PUFAs and PhenoAgeAccel. The RCS models with three knots (at the 10th, 50th, and 90th percentiles) were used to explore the potential nonlinear relationship between PhenoAge acceleration and log10-transformed PUFAs.

Mediation analyses were performed to investigate the role of SII as a mediator in the relationship between serum PUFAs and PhenoAge acceleration. The direct effect (DE) quantified the impact of serum PUFAs on PhenoAge acceleration without the involvement of a mediator, while the indirect effect (IE) measured the influence of serum PUFAs on PhenoAge acceleration through the mediation of SII. The proportion of the mediation effect was determined by dividing the IE by the total effect. WQS and QGC model was applied to evaluate the combined effects of serum PUFAs and identify major fatty acids. In WQS model, data was randomly split into training sets (40%) and validation sets (60%) and weight estimations were derived from 1000 bootstrapped samples. Due to the unidirectional characteristic of WQS, we applied the models to PUFAs in both negative and positive directions. Fatty acids were identified as critical if their absolute weight exceeded the average. In QGC model, the overall association was assessed as the joint effect of a one-quartile increase in mixed PUFAs on PhenoAge acceleration. The QGC model was conducted without bootstrap for estimating the weight of each serum PUFA and with bootstrap set to 1000 for estimating the marginal odds ratio of the joint effect. The weights of each PUFA represented the proportion of negative or positive contributions to the mixture exposure, with the range from − 1 to 1. Sensitivity analyses were conducted to further assess the results. Firstly, the association between PUFAs and PhenoAge acceleration was reanalyzed without accounting for complex sampling design. Secondly, weighted multivariate logistic regression was performed based on the presence or absence of accelerated aging. Thirdly, to evaluate the impact of PUFAs on all-cause and CVD-related mortality, weighted multivariate Cox regression was conducted. Statistical significance was defined as a two-tailed *P* value < 0.05. All analyses were conducted in R (version 4.3.2). The following R packages were used: “survey”, “rms”, “mediation”, “gWQS,” and “Qgcomp.”

## Results

### Baseline characteristics of the participants

From 2011 to 2014, a cohort of 3376 participants was enrolled in this study. Table 1 presented the baseline characteristics of participants, stratified by the presence of accelerated aging. The median Phenoage acceleration year for participants with accelerated aging was 3.0 years (IQR: 1.2, 5.7), which was significantly higher than that of those without accelerated aging (− 4.5 years, IQR: − 6.7, − 2.5). Compared to the non-accelerated aging group, individuals with accelerated aging were more likely to be older, male, non-Hispanic Black, have a lower education level, a lower poverty income ratio, higher BMI, and a history of smoking, diabetes, hypertension, cardiovascular disease, and cancer. Serum levels of total n-6 PUFAs and n-3 PUFAs were higher in the non-accelerated aging group. Specifically, LA, ALA, SDA, EPA, DHA were significantly higher.

**Table 1:** Baseline characteristics of participants

Variable	Overall *N* = 165,127,735, *n* = 3376 (100.0%)^1^	Accelerated aging *N* = 43,474,217, *n* = 1000 (26.3%)^1^	Non-accelerated aging *N* = 121,653,518, *n* = 2376 (73.7%)^1^	*p*-value^2^
Age, years	46.0 (33.0, 60.0)	48.0 (35.0, 64.0)	45.0 (32.0, 59.0)	< 0.001
Sex				0.044
Male	1639 (49.2%)	536 (53.3%)	1103 (47.7%)	
Female	1737 (50.8%)	464 (46.7%)	1273 (52.3%)	
Race/ethnicity				< 0.001
Non-Hispanic white	1505 (68.9%)	466 (66.1%)	1039 (69.9%)	
Non-Hispanic black	663 (10.6%)	267 (16.4%)	396 (8.5%)	
Mexican American	711 (13.6%)	190 (12.9%)	521 (13.9%)	
Others	497 (6.9%)	77 (4.6%)	420 (7.7%)	
Education level				< 0.001
Less than high School	691 (15.2%)	240 (20.0%)	451 (13.5%)	
High school or equivalent	704 (19.3%)	242 (23.4%)	462 (17.8%)	
College or above	1981 (65.5%)	518 (56.6%)	1463 (68.6%)	
Poverty income ratio				< 0.001
< = 1.3	1139 (24.1%)	417 (33.0%)	722 (20.8%)	
1.3–3.5	1161 (33.7%)	338 (34.6%)	823 (33.4%)	
> 3.5	1076 (42.3%)	245 (32.4%)	831 (45.8%)	
Body Mass Index (kg/m2)				< 0.001
Under/normal	1056 (30.2%)	223 (22.4%)	833 (33.0%)	
Overweight	1112 (34.2%)	291 (27.5%)	821 (36.6%)	
Obesity	1208 (35.6%)	486 (50.2%)	722 (30.4%)	
Smoking status				< 0.001
Never	1945 (57.6%)	436 (42.8%)	1509 (63.0%)	
Former	767 (23.3%)	247 (25.5%)	520 (22.5%)	
Current	664 (19.0%)	317 (31.7%)	347 (14.5%)	
Diabetes	388 (8.6%)	243 (20.5%)	145 (4.4%)	< 0.001
Hypertension	1214 (33.2%)	482 (44.4%)	732 (29.3%)	< 0.001
Cardiovascular disease	333 (8.3%)	184 (16.5%)	149 (5.4%)	< 0.001
Cancer	302 (9.7%)	121 (11.4%)	181 (9.0%)	0.11
Total n-6 PUFAs (umol/L)	4655.3 (4006.4, 5375.7)	4545.2 (3825.1, 5370.4)	4698.4 (4083.9, 5375.7)	0.035
LA (umol/L)	3530.0 (2990.0, 4080.0)	3390.0 (2830.0, 4030.0)	3580.0 (3040.0, 4120.0)	0.006
GLA (umol/L)	53.4 (37.7, 76.4)	55.7 (39.2, 78.8)	52.3 (37.0, 75.3)	0.042
EDA (umol/L)	21.4 (17.3, 26.8)	21.8 (17.2, 27.9)	21.3 (17.4, 26.4)	0.5
HGLA (umol/L)	156.0 (121.0, 196.0)	158.0 (118.0, 203.0)	156.0 (122.0, 195.0)	0.6
AA (umol/L)	830.0 (685.0, 1010.0)	843.0 (699.0, 1030.0)	828.0 (680.0, 1000.0)	0.2
DTA (umol/L)	25.5 (20.0, 32.7)	28.2 (22.0, 34.9)	24.7 (19.5, 31.6)	< 0.001
n-6 DPA (umol/L)	19.3 (14.5, 25.3)	20.4 (15.8, 26.5)	18.8 (14.1, 24.9)	< 0.001
Total n-3 PUFAs (umol/L)	333.4 (262.9, 434.3)	322.9 (251.5, 408.6)	339.1 (265.4, 442.1)	0.021
ALA (umol/L)	76.7 (56.5, 107.0)	74.1 (54.4, 110.0)	77.0 (57.9, 105.0)	0.5
SDA (umol/L)	3.1 (1.9, 5.0)	3.1 (2.0, 5.0)	3.1 (1.9, 5.0)	0.7
EPA (umol/L)	51.8 (35.3, 79.8)	47.8 (33.4, 72.8)	53.0 (36.2, 82.1)	0.003
n-3 DPA (umol/L)	49.2 (39.3, 62.5)	49.2 (39.1, 61.5)	49.2 (39.4, 62.9)	0.4
DHA (umol/L)	140.0 (106.0, 192.0)	131.0 (97.7, 179.0)	144.0 (109.0, 196.0)	0.005
n-6/n-3	14.2 (11.5, 16.8)	14.6 (11.9, 16.8)	14.1 (11.3, 16.8)	0.12
PhenoAge, years	43.4 (29.7, 57.3)	52.1 (38.8, 67.1)	39.9 (27.0, 53.8)	< 0.001
PhenoAge Acceleration, years	− 3.1 (− 6.0, 0.2)	3.0 (1.2, 5.7)	− 4.5 (− 6.7, − 2.5)	< 0.001

^1^Median (Q1, Q3); n (unweighted) (%); N (weighted) (%)

^2^Design-based KruskalWallis test; Pearson's X^2

### Association between serum PUFAs and PhenoAge acceleration

The detailed results of weighted multivariate linear regression analysis were presented in Tables 2 and 3. Among the serum n-6 PUFAs, total n-6 and LA exhibited significant negative associations with PhenoAgeAccel across all three models, whereas GLA and AA showed a negatively relationship only in model 3. DTA and n-6 DPA showed a positive relationship in Model 1 and Model 2, but no significant association in Model 3 after adjusting for additional confounding covariates. In the fully adjusted model (Model 3), each SD increment in total n-6, LA, GLA, and AA were associated with reductions in PhenoAge acceleration of − 0.4382 (95%CI − 0.7030 to − 0.1733, *P* = 0.003), − 0.4495 (95%CI − 0.7141 to − 0.1850, *P* = 0.003), − 0.2823 (95%CI − 0.4699 to − 0.0946, *P* = 0.006), and − 0.2969 (95%CI − 0.5155 to − 0.0782, *P* = 0.011), respectively. When comparing participants in the highest tertile (Q3) to those in the lowest tertile (Q1, reference), PhenoAgeAccel was significantly lower by − 1.1211 (95%CI − 1.6338 to − 0.6084, *P* < 0.001) for total n-6, − 0.9576 (95%CI − 1.5180 to − 0.3972, *P* = 0.003) for LA, − 0.5467 (95%CI − 0.9461 to − 0.1473, *P* = 0.011) for GLA, and − 0.6915 (95%CI − 1.1978 to − 0.1852, *P* = 0.011) for AA. Among the serum n-3 PUFAs, total n-3 and its subtypes ALA, SDA, EPA, n-3 DPA, and DHA were all significantly inversely associated with PhenoAgeAccel in Model 3. In contrast, the n-6 to n-3 ratio demonstrated a positive relationship in Model 1 and Model 2, but this association was no longer significant in Model 3. Each SD increase in total n-3, ALA, SDA, EPA, n-3 DPA, and DHA was linked to a decrease in PhenoAge acceleration, with reductions of − 0.5101 (95%CI − 0.7700 to − 0.2503, *P* < 0.001), − 0.2835 (95%CI − 0.5234 to − 0.0435, *P* = 0.024), − 0.3731 (95%CI − 0.5687 to − 0.1776, *P* = 0.001), − 0.3815 (95%CI − 0.6423 to − 0.1208, *P* = 0.007), − 0.4212 (95%CI − 0.6364 to − 0.2059, *P* < 0.001), − 0.5083 (95%CI − 0.7850 to − 0.2316, *P* = 0.001), respectively. Compared to participants in the lowest tertile (Q1), those in the highest tertile (Q3) of total n-3, ALA, SDA, EPA, n-3 DPA, and DHA exhibited a greater reduction in PhenoAgeAccel: − 1.1108 (95%CI − 1.6231 to − 0.5985, *P* < 0.001), − 0.9206 (95%CI − 1.5055 to − 0.3357, *P* = 0.005), − 0.7355 (95%CI − 1.1246 to − 0.3464, *P* = 0.001), − 0.9866 (95%CI − 1.5805 to − 0.3926, *P* = 0.003), − 0.8993 (95%CI − 1.3968 to − 0.4018, *P* = 0.002), and − 1.1276 (95%CI − 1.7735 to − 0.4818, *P* = 0.002), respectively.

**Table 2:** Weighted multivariate linear regression analysis of serum n-6 PUFAs with PhenoAgeAccel

	Model 1		Model 2		Model 3	
	β (95% CI)	p	β (95% CI)	p	β (95% CI)	p
Total n-6 (continuous)	– 0.0004 (– 0.0006, – 0.0001)	0.003	– 0.0003 (– 0.0006, – 0.0001)	0.005	– 0.0004 (– 0.0006, – 0.0001)	0.003
Total n-6 (per SD)	– 0.4124 (– 0.6754, – 0.1494)	0.003	– 0.3974 (– 0.6671, – 0.1277)	0.005	– 0.4382 (– 0.7030, – 0.1733)	0.003
Tertile 1	ref		ref		ref	
Tertile 2	– 1.3250 (– 1.8428, – 0.8072)	< 0.001	– 1.2471 (– 1.7625, – 0.7317)	< 0.001	– 1.0103 (– 1.4766, – 0.5441)	< 0.001
Tertile 3	– 1.1493 (– 1.6450, – 0.6537)	< 0.001	– 1.1141 (– 1.6020, – 0.6263)	< 0.001	– 1.1211 (– 1.6338, – 0.6084)	< 0.001
*p* for trend		< 0.001		< 0.001		< 0.001
LA (continuous)	– 0.0006 (– 0.0008, – 0.0003)	< 0.001	– 0.0005 (– 0.0008, – 0.0002)	0.002	– 0.0005 (– 0.0007, – 0.0002)	0.003
LA (per SD)	– 0.5493 (– 0.8265, – 0.2720)	< 0.001	– 0.4850 (– 0.7686, – 0.2013)	0.002	– 0.4495 (– 0.7141, – 0.1850)	0.003
Tertile 1	ref		ref		ref	
Tertile 2	– 1.3410 (– 1.9076, – 0.7743)	< 0.001	– 1.1506 (– 1.7109, – 0.5903)	< 0.001	– 0.7287 (– 1.3003, – 0.1571)	0.016
Tertile 3	– 1.3394 (– 1.9246, – 0.7541)	< 0.001	– 1.1408 (– 1.7132, – 0.5683)	< 0.001	– 0.9576 (– 1.5180, – 0.3972)	0.003
*p* for trend		< 0.001		< 0.001		0.003
GLA (continuous)	0.0022 (– 0.0034, 0.0077)	0.430	– 0.0003 (– 0.0054, 0.0048)	0.904	– 0.0087 (– 0.0145, – 0.0029)	0.006
GLA (per SD)	0.0702 (– 0.1088, 0.2492)	0.430	– 0.0098 (– 0.1753, 0.1556)	0.904	– 0.2823 (– 0.4699, – 0.0946)	0.006
Tertile 1	ref		ref		ref	
Tertile 2	0.2406 (– 0.3987, 0.8799)	0.448	– 0.0501 (– 0.7058, 0.6056)	0.876	– 0.4521 (– 0.9814, 0.0773)	0.088
Tertile 3	0.4490 (0.0532, 0.8447)	0.028	0.1699 (– 0.2634, 0.6031)	0.427	– 0.5467 (– 0.9461, – 0.1473)	0.011
*p* for trend		0.034		0.354		0.024
EDA (continuous)	0.0053 (– 0.0266, 0.0371)	0.739	0.0110 (– 0.0226, 0.0445)	0.507	– 0.0053 (– 0.0381, 0.0275)	0.732
EDA (per SD)	0.0468 (– 0.2371, 0.3307)	0.739	0.0979 (– 0.2011, 0.3968)	0.507	– 0.0475 (– 0.3396, 0.2445)	0.732
Tertile 1	ref		ref		ref	
Tertile 2	– 0.5132 (– 0.9864, – 0.0399)	0.035	– 0.5081 (– 0.9460, – 0.0703)	0.025	– 0.6281 (– 1.0462, – 0.2100)	0.006
Tertile 3	0.0137 (– 0.5818, 0.6092)	0.963	0.1305 (– 0.4696, 0.7306)	0.658	– 0.2240 (– 0.8173, 0.3692)	0.429
*p* for trend		0.831		0.531		0.566
HGLA (continuous)	0.0001 (– 0.0037, 0.0038)	0.976	0.0018 (– 0.0021, 0.0056)	0.351	– 0.0020 (– 0.0059, 0.0020)	0.302
HGLA (per SD)	0.0034 (– 0.2241, 0.2309)	0.976	0.1060 (– 0.1236, 0.3356)	0.351	– 0.1187 (– 0.3564, 0.1190)	0.302
Tertile 1	ref		ref		ref	
Tertile 2	– 0.5129 (– 0.9921, – 0.0336)	0.037	– 0.3998 (– 0.8687, 0.0691)	0.091	– 0.3615 (– 0.7925, 0.0694)	0.093
Tertile 3	– 0.1470 (– 0.7368, 0.4427)	0.614	0.0878 (– 0.5261, 0.7017)	0.771	– 0.3944 (– 1.0170, 0.2282)	0.194
*p* for trend		0.776		0.623		0.241
AA (continuous)	0.0003 (– 0.0005, 0.0011)	0.445	– 0.0005 (– 0.0013, 0.0004)	0.271	– 0.0012 (– 0.0020, – 0.0003)	0.011
AA (per SD)	0.0791 (– 0.1295, 0.2878)	0.445	– 0.1151 (– 0.3254, 0.0952)	0.271	– 0.2969 (– 0.5155, – 0.0782)	0.011
Tertile 1	ref		ref		ref	
Tertile 2	0.1944 (– 0.2567, 0.6456)	0.386	– 0.1505 (– 0.6268, 0.3258)	0.521	– 0.2536 (– 0.7013, 0.1942)	0.243
Tertile 3	0.1054 (– 0.4306, 0.6414)	0.691	– 0.3780 (– 0.9202, 0.1641)	0.163	– 0.6915 (– 1.1978, – 0.1852)	0.011
*p* for trend		0.725		0.159		0.010
DTA (continuous)	0.0692 (0.0454, 0.0930)	< 0.001	0.0595 (0.0376, 0.0814)	< 0.001	0.0192 (– 0.0046, 0.0431)	0.106
DTA (per SD)	0.7595 (0.4982, 1.0208)	< 0.001	0.6533 (0.4131, 0.8934)	< 0.001	0.2110 (– 0.0508, 0.4728)	0.106
Tertile 1	ref		ref		ref	
Tertile 2	1.1355 (0.6426, 1.6283)	< 0.001	0.9454 (0.4560, 1.4347)	< 0.001	0.3499 (– 0.0978, 0.7976)	0.115
Tertile 3	1.7816 (1.1952, 2.3681)	< 0.001	1.5208 (0.9660, 2.0757)	< 0.001	0.5732 (0.0091, 1.1373)	0.047
*p* for trend		< 0.001		< 0.001		0.050
n-6 DPA (continuous)	0.0509 (0.0227, 0.0790)	< 0.001	0.0522 (0.0245, 0.0799)	< 0.001	0.0191 (– 0.0103, 0.0484)	0.185
n-6 DPA (per SD)	0.4686 (0.2095, 0.7277)	< 0.001	0.4810 (0.2256, 0.7364)	< 0.001	0.1756 (– 0.0946, 0.4458)	0.185
Tertile 1	ref		ref		ref	
Tertile 2	0.6123 (– 0.0172, 1.2418)	0.056	0.5913 (0.0047, 1.1780)	0.048	0.2163 (– 0.1694, 0.6021)	0.247
Tertile 3	0.8066 (0.2040, 1.4093)	0.010	0.7524 (0.1700, 1.3349)	0.013	0.0902 (– 0.4589, 0.6393)	0.728
*p* for trend		0.012		0.016		0.771

Model 1: Unadjusted. Model 2: Adjusted for age, sex, and race/ethnicity. Model 3: Adjusted for age, sex, race/ethnicity, education, poverty income ratio, body mass index, smoking status, diabetes, hypertension, cardiovascular disease, cancer. CI: Confidence interval; ref: reference level; PhenoAgeAceel: phenotypic age acceleration

**Table 3:** Weighted multivariate linear regression analysis of serum n-3 PUFAs with PhenoAgeAccel

	Model 1		Model 2		Model 3	
	β (95% CI)	p	β (95% CI)	p	β (95% CI)	p
Total n-3 (continuous)	− 0.0031 (− 0.0048, − 0.0014)	< 0.001	− 0.0037 (− 0.0056, − 0.0018)	< 0.001	− 0.0028 (− 0.0043, − 0.0014)	< 0.001
Total n-3 (per SD)	− 0.5588 (− 0.8723, − 0.2454)	< 0.001	− 0.6669 (− 1.0084, − 0.3254)	< 0.001	− 0.5101 (− 0.7700, − 0.2503)	< 0.001
Tertile 1	ref		ref		ref	
Tertile 2	− 0.3852 (− 0.9492, 0.1787)	0.173	− 0.5540 (− 1.0483, − 0.0598)	0.030	− 0.2860 (− 0.7635, 0.1915)	0.218
Tertile 3	− 1.3773 (− 2.0152, − 0.7394)	< 0.001	− 1.6486 (− 2.3182, − 0.9790)	< 0.001	− 1.1108 (− 1.6231, − 0.5985)	< 0.001
*p* for trend		< 0.001		< 0.001		< 0.001
ALA (continuous)	− 0.0037 (− 0.0077, 0.0004)	0.075	− 0.0031 (− 0.0075, 0.0013)	0.161	− 0.0050 (− 0.0093, − 0.0008)	0.024
ALA (per SD)	− 0.2065 (− 0.4352, 0.0222)	0.075	− 0.1734 (− 0.4202, 0.0733)	0.161	− 0.2835 (− 0.5234, − 0.0435)	0.024
Tertile 1	ref		ref		ref	
Tertile 2	− 1.1247 (− 1.6457, − 0.6037)	< 0.001	− 1.0353 (− 1.5534, − 0.5172)	< 0.001	− 0.8968 (− 1.3772, − 0.4164)	0.001
Tertile 3	− 0.8214 (− 1.4649, − 0.1779)	0.014	− 0.7401 (− 1.4233, − 0.0568)	0.035	− 0.9206 (− 1.5055, − 0.3357)	0.005
*p* for trend		0.037		0.077		0.007
SDA (continuous)	− 0.0478 (− 0.1085, 0.0129)	0.118	− 0.0612 (− 0.1243, 0.0018)	0.057	− 0.1139 (− 0.1736, − 0.0542)	0.001
SDA (per SD)	− 0.1566 (− 0.3556, 0.0423)	0.118	− 0.2007 (− 0.4074, 0.0060)	0.057	− 0.3731 (− 0.5687, − 0.1776)	0.001
Tertile 1	ref		ref		ref	
Tertile 2	− 0.1293 (− 0.7352, 0.4765)	0.666	− 0.3248 (− 0.9365, 0.2868)	0.284	− 0.6622 (− 1.1646, − 0.1597)	0.014
Tertile 3	− 0.1017 (− 0.5497, 0.3464)	0.646	− 0.2694 (− 0.7314, 0.1927)	0.241	− 0.7355 (− 1.1246, − 0.3464)	0.001
*p* for trend		0.745		0.394		0.007
EPA (continuous)	− 0.0073 (− 0.0122, − 0.0025)	0.004	− 0.0094 (− 0.0146, − 0.0041)	0.001	− 0.0064 (− 0.0107, − 0.0020)	0.007
EPA (per SD)	− 0.4393 (− 0.7311, − 0.1475)	0.004	− 0.5620 (− 0.8790, − 0.2451)	0.001	− 0.3815 (− 0.6423, − 0.1208)	0.007
Tertile 1	ref		ref		ref	
Tertile 2	− 0.4463 (− 0.9361, 0.0434)	0.073	− 0.7018 (− 1.1790, − 0.2246)	0.006	− 0.5029 (− 0.8862, − 0.1197)	0.014
Tertile 3	− 1.0962 (− 1.7166, − 0.4759)	0.001	− 1.4421 (− 2.1061, − 0.7781)	< 0.001	− 0.9866 (− 1.5805, − 0.3926)	0.003
*p* for trend		< 0.001		< 0.001		0.004
n-3 DPA (continuous)	− 0.0101 (− 0.0203, − 0.0000)	0.050	− 0.0177 (− 0.0292, − 0.0063)	0.004	− 0.0206 (− 0.0312, − 0.0101)	< 0.001
n-3 DPA (per SD)	− 0.2071 (− 0.4137, − 0.0004)	0.050	− 0.3620 (− 0.5960, − 0.1279)	0.004	− 0.4212 (− 0.6364, − 0.2059)	< 0.001
Tertile 1	ref		ref		ref	
Tertile 2	0.0173 (− 0.4768, 0.5114)	0.944	− 0.2557 (− 0.7804, 0.2690)	0.325	− 0.3723 (− 0.8040, 0.0593)	0.085
Tertile 3	− 0.4913 (− 0.9493, − 0.0334)	0.036	− 0.8612 (− 1.3628, − 0.3596)	0.002	− 0.8993 (− 1.3968, − 0.4018)	0.002
*p* for trend		0.025		0.001		0.001
DHA (continuous)	− 0.0078 (− 0.0118, − 0.0039)	< 0.001	− 0.0094 (− 0.0137, − 0.0051)	< 0.001	− 0.0058 (− 0.0090, − 0.0026)	0.001
DHA (per SD)	− 0.6863 (− 1.0327, − 0.3400)	< 0.001	− 0.8179 (− 1.1941, − 0.4417)	< 0.001	− 0.5083 (− 0.7850, − 0.2316)	0.001
Tertile 1	ref		ref		ref	
Tertile 2	− 0.9215 (− 1.4986, − 0.3444)	0.003	− 1.1359 (− 1.6894, − 0.5823)	< 0.001	− 0.8656 (− 1.3916, − 0.3395)	0.004
Tertile 3	− 1.6441 (− 2.4146, − 0.8735)	< 0.001	− 1.9369 (− 2.7431, − 1.1308)	< 0.001	− 1.1276 (− 1.7735, − 0.4818)	0.002
*p* for trend		< 0.001		< 0.001		0.005
n-6/n-3 (continuous)	0.0861 (0.0058, 0.1663)	0.036	0.1239 (0.0290, 0.2189)	0.012	0.0636 (− 0.0150, 0.1421)	0.105
n-6/n-3 (per SD)	0.3478 (0.0234, 0.6723)	0.036	0.5009 (0.1173, 0.8845)	0.012	0.2569 (− 0.0606, 0.5744)	0.105
Tertile 1	ref		ref		ref	
Tertile 2	0.7189 (0.0713, 1.3666)	0.031	0.9159 (0.2253, 1.6065)	0.011	0.4413 (− 0.2117, 1.0942)	0.168
Tertile 3	0.9147 (0.1952, 1.6343)	0.014	1.2248 (0.3998, 2.0498)	0.005	0.5957 (− 0.0918, 1.2832)	0.084
*p* for trend		0.016		0.006		0.087

Model 1: Unadjusted. Model 2: Adjusted for age, sex, and race/ethnicity. Model 3: Adjusted for age, sex, race/ethnicity, education, poverty income ratio, body mass index, smoking status, diabetes, hypertension, cardiovascular disease, cancer. CI: Confidence interval; ref: reference level; PhenoAgeAceel: phenotypic age acceleration

**Fig. 1:**
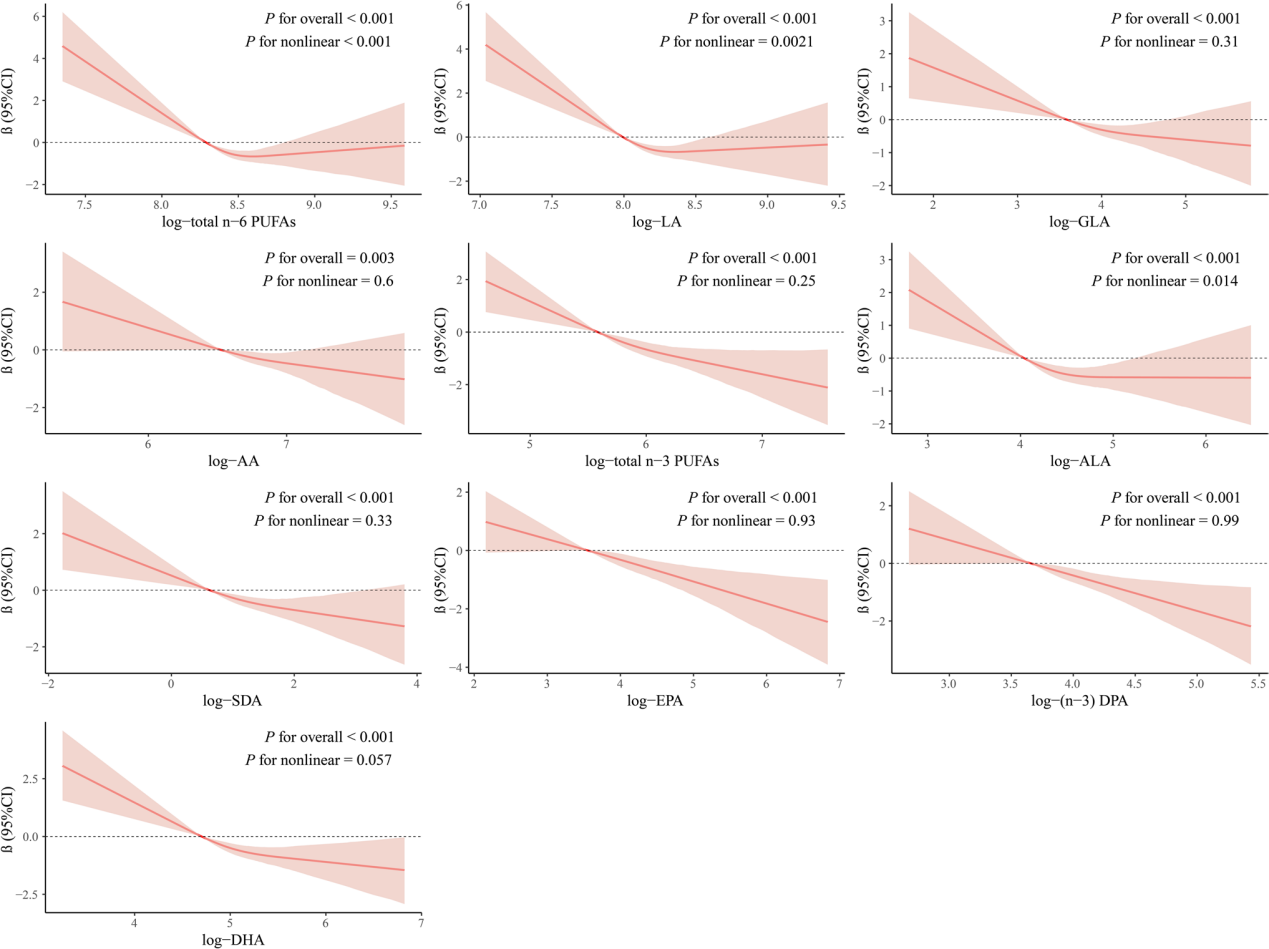
Restricted cubic splines analysis for the association between log-transformed serum PUFAs and PhenoAge acceleration. The solid lines represent the estimates of effects and the shaded area represents the 95% confidence interval. The model was adjusted for age, sex, race/ethnicity, education, poverty income ratio, body mass index, smoking status, diabetes, hypertension, cardiovascular disease, and cancer

Furthermore, RCS analyses was used to explore the dose–response relationship between the log10-transformed serum PUFAs and PhenoAgeAccel, as shown in Fig. 1. We observed negative linear relationships between GLA, AA, total n-3, SDA, EPA, n-3 DPA, and DHA with PhenoAgeAccel (all *P for nonlinear* > 0.05). An L-shaped relationship was found between total n-6 PUFAs, LA, and ALA and PhenoAge acceleration (all *P for nonlinear* < 0.05).

### Mixture effects of multiple PUFAs on PhenoAge acceleration

**Fig. 2:**
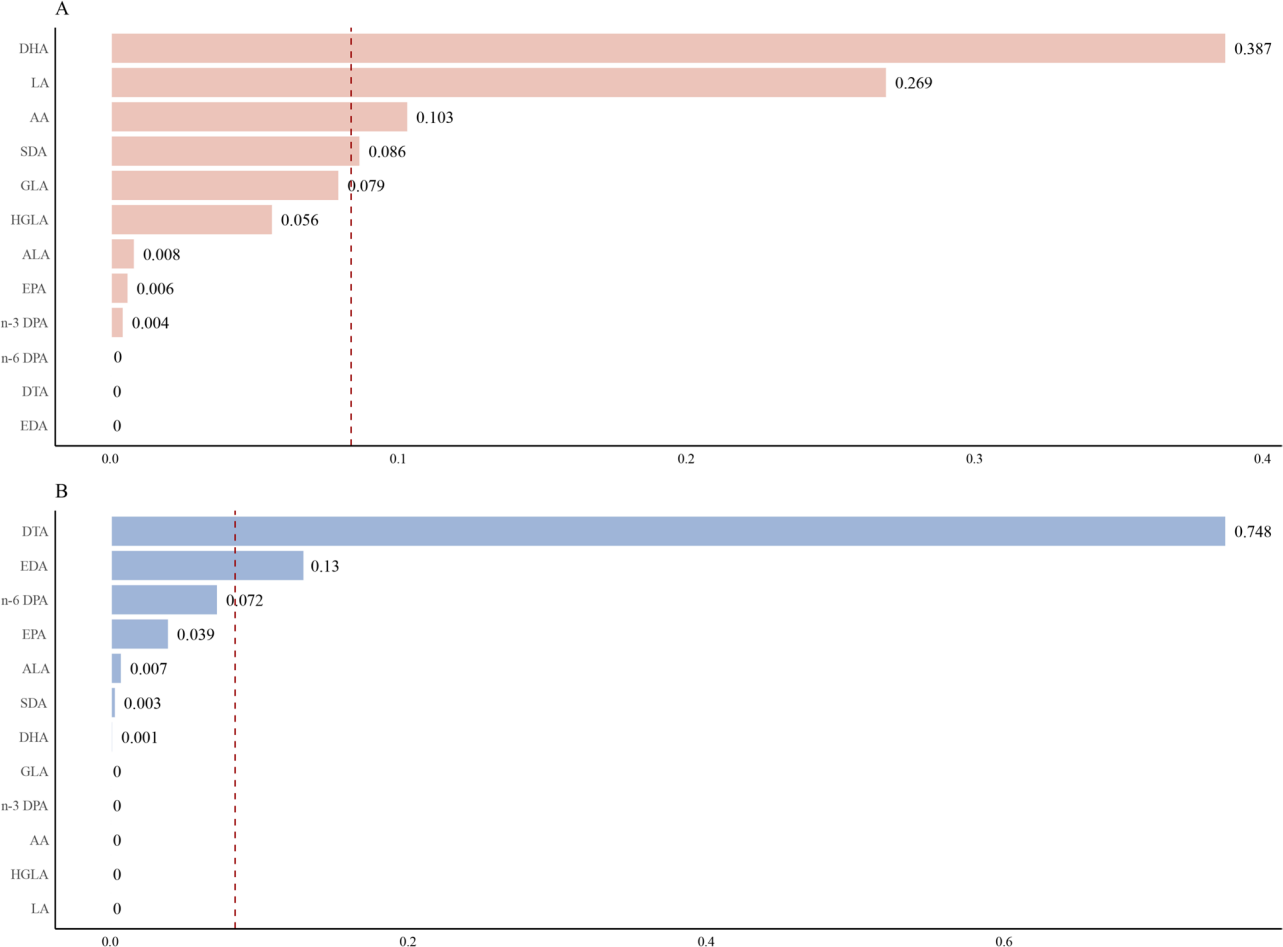
The weights of positive and negative effects of serum PUFAs on PhenoAge acceleration in the WQS model. **a** the negative WQS model. **b** the positive WQS model. The dashed line indicated the average weight, 1/n, where n is the number of PUFAs analyzed. WQS: weighted quantile sum

As illustrated in Fig. 2, the WQS model examined the relationship between the PUFAs mixtures and PhenoAge acceleration in both negative and positive directions. In the negative constraint models, the combined effect of PUFAs was inversely associated with the PhenoAge acceleration (*β* = − 0.761, 95% CI − 1.036 to − 0.490, *P* < 0.001). DHA (38.7%) was identified as the most critical contributors, followed by LA (26.9%), AA (10.3%), SDA (8.6%). When the model was constrained in the positive direction, DHA and EDA contributed weighted values of 74.8% and 13%, respectively, to the positive mixture effect.

**Fig. 3:**
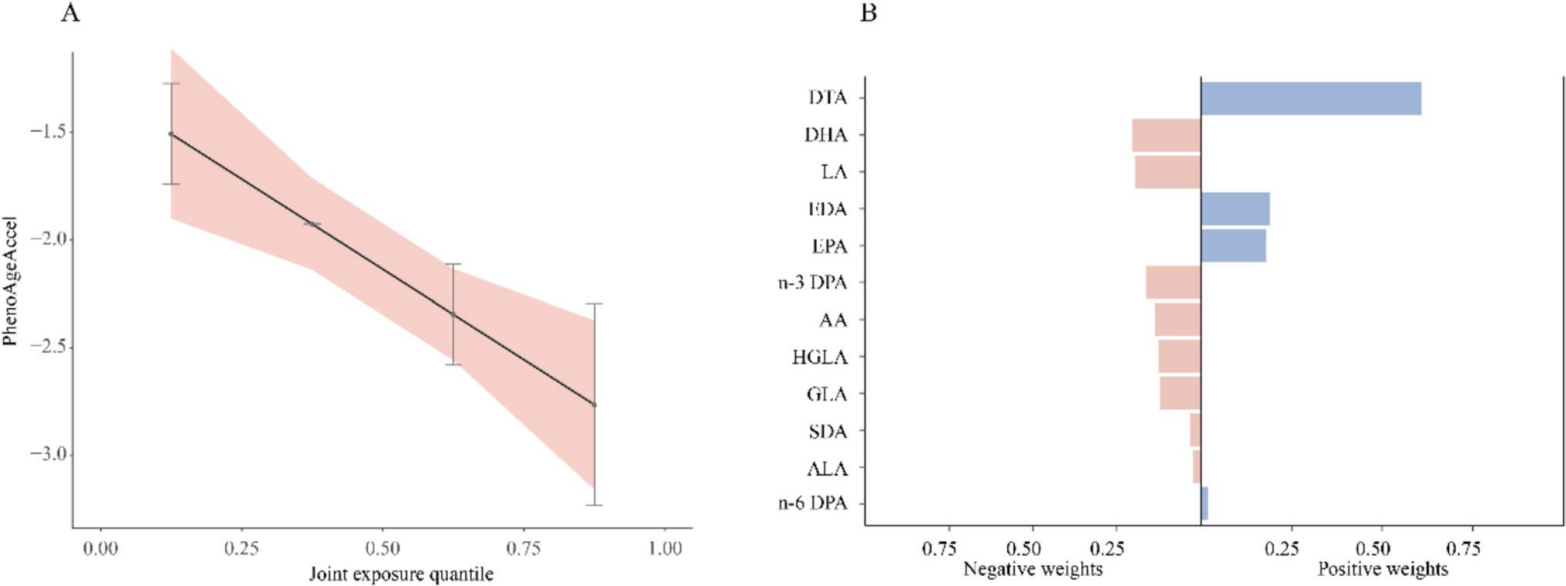
The weights of positive and negative effects of serum PUFAs on PhenoAge acceleration and the combined effects of mixed PUFAs to PhenoAge acceleration in the QGC model. **a** The effect of mixed PUFAs; **b** the proportion of the positive or negative effects for each PUFAs; QGC: quantile g-computation

Subsequently, we used the QGC model to further assess the mixture effects of PUFAs. As shown in Fig. 3a, an increase in each quartile of the mixed PUFAs was negatively associated with PhenoAge acceleration (*β* = − 0.420, 95% CI − 0.656 to − 0.185, *P* < 0.001). Figure 3b illustrated the distribution of individual PUFA weights. The key negative weights of PUFAs were contributed by DHA (20.4%), and the positive weight was predominantly attributed to DTA (60.8%).

### Mediation effects of SII in the relationship of individual PUFAs with PhenoAge acceleration

**Fig. 4:**
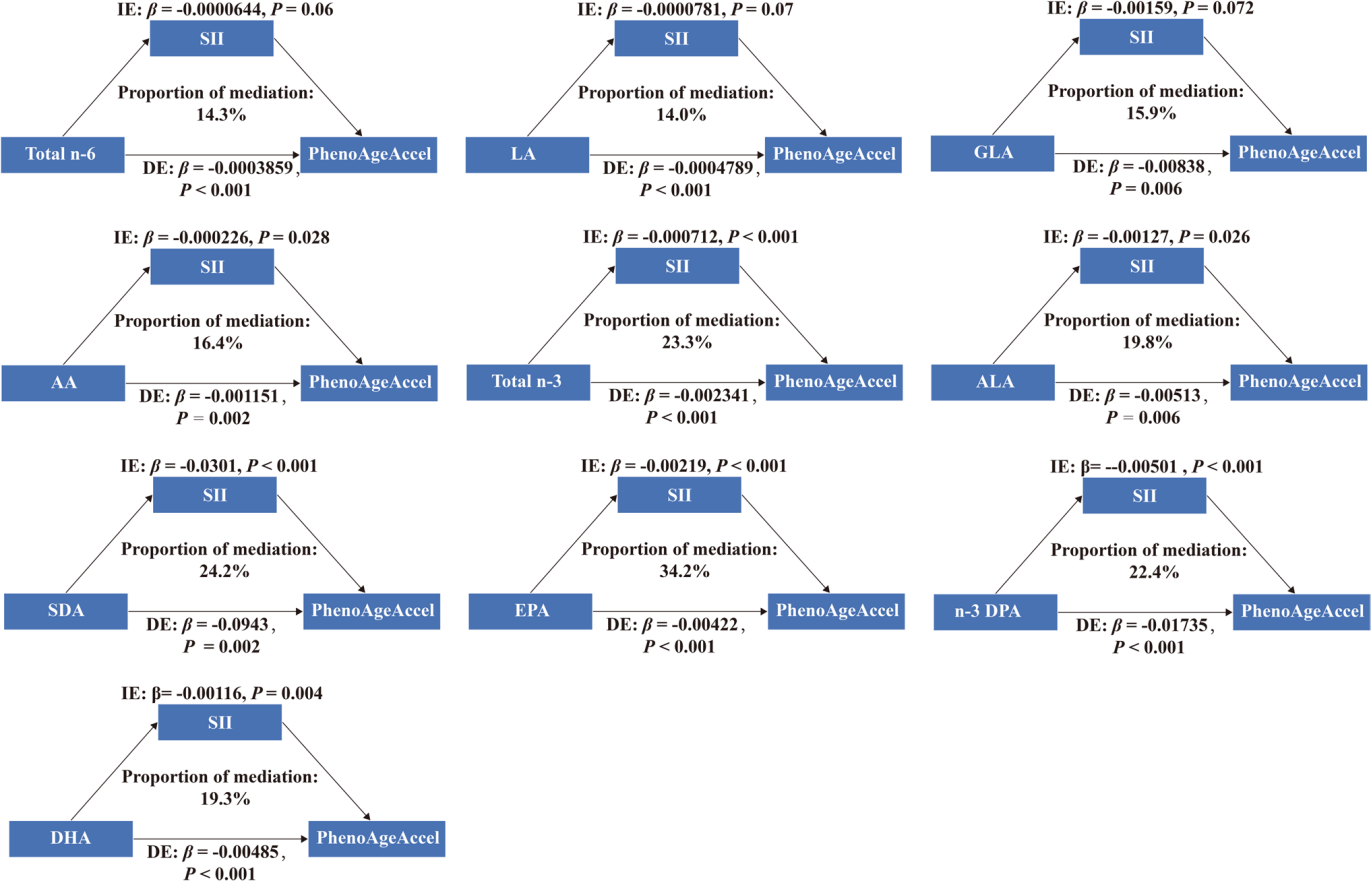
The mediating proportion of SII on the association between serum n-6 and n-3 PUFAs and PhenoAge acceleration. Model was adjusted for age, sex, race/ethnicity, education, poverty income ratio, body mass index, smoking status, diabetes, hypertension, cardiovascular disease, cancer. SII, systemic immune-inflammation index; IE: indirect effects; DE: direct effects

Significant mediating effects of SII were observed in the associations between PUFAs and PhenoAge acceleration, except for total n-6 PUFAs, LA, and GLA (Fig. 4). The proportion of the mediation effect of SII in the relationships between AA, total n-3 PUFAs, ALA, SDA, EPA, n-3 DPA, and DHA with PhenoAge acceleration was 16.4%, 23.3%, 19.8%, 24.2%, 34.2%, 22.4%, and 19.3%, respectively (all *P* < 0.05).

### Subgroup and sensitivity analyses

To explore the potential effects of interactive factors, we conducted subgroup analyses by age, sex, and race/ethnicity. The results were presented in Supplementary Figs. 3–5. These analyses revealed the association between serum total n-6, LA, GLA, AA, total n-3, ALA, SDA, EPA, n-3 DPA, and DHA and PhenoAge acceleration were consistently robust across different age, sex and race/ethnicity subgroups (all *P for interaction* > 0.05). Sensitivity analyses were conducted to validate the findings. When excluding complex sampling design (Supplementary Table 1), the results of the linear regression model confirmed the main findings. Additionally, When PhenoAge acceleration was categorized based on the presence of accelerated aging, weighted logistic regression results showed that, compared to the lowest quartile, participants in higher quartiles of LA, total n-3 PUFAs, EPA, and DHA were associated with a reduced risk of accelerated aging (Supplementary Table 2). Given the prominent role of DHA in these findings, we also performed weighted Cox regression analysis to assess the associations between PUFAs and all-cause and CVD-related mortality (Supplementary Tables 3–4). The results indicated that participants in higher quartiles of DHA had a lower risk of all-cause mortality compared to those in the lowest quartile.

## Discussion

To our knowledge, this is the first study to explore the association between serum levels of omega-3 and omega-6 PUFAs and PhenoAge acceleration, as well as the first to investigate the mediating role of the SII in this relationship. We employed a range of statistical methods, including weighted multivariate linear regression, subgroup analysis, RCS, WQS, and QGC analyses to assess both the individual and combined effects of PUFAs. Mediation analyses were also applied to investigate the role of SII in these relationships. In this study, our results revealed that total n-6 PUFAs, total n-3 PUFAs, three n-6 subtypes (LA, GLA and AA) and five n-3 subtypes (ALA, SDA, EPA, n-3 DPA and DHA) were negatively correlated with PhenoAge acceleration. Conversely, no significant correlation was observed between EDA, HGLA, DTA and n-6 DPA and PhenoAge acceleration in the adjusted model. These associations were robust and consistent across various subgroups, including age, sex, and race/ethnicity. Notably, L-shaped non-linear relationships were observed for total n-6 PUFAs, LA, and ALA with PhenoAge acceleration. Mediation analysis confirmed that SII significantly mediated the associations between AA, total n-3 PUFAs, ALA, SDA, EPA, n-3 DPA, and DHA and PhenoAge acceleration. Furthermore, mixed-effect analyses revealed that the overall effects of PUFAs were negatively correlated with PhenoAge acceleration in both WQS and QGC analyses. DHA was identified as the primary contributor to the negative association with PhenoAge acceleration. These findings contribute to a deeper understanding of the mechanisms of biological aging, highlighting the potential role of PUFAs in modulating PhenoAge acceleration.

In our study, serum PUFAs levels were measured as a reliable and objective indicator of dietary fat composition, which has been shown to provide insights into how individuals biologically respond to their fat intake patterns [15]. Several studies have suggested that long-chain polyunsaturated fatty acids (LC-PUFAs) may be a potentially important contributor to slowing accelerated aging, as well as delaying the onset of age-related diseases. Moreover, some studies have identified an association between LC-PUFAs and various markers of biological aging, including telomere length, DNA methylation, and age-related diseases such as cognitive decline, and frailty. Telomere elongation is often regarded as an indicator of biological longevity. In a recent study, Magdalena et al. utilized a transgenic murine model that expresses the fat-1 gene, which increases n-3 fatty acid levels and reduces the n-6 to n-3 ratio. The study found that increased levels of endogenous n-3 fatty acids were correlated with longer telomere length [16]. Similarly, Varinderpal et al. investigated the relationship between serum PUFAs and telomere length, observing a positive correlation with longer telomeres in their study of healthy adults [17]. In addition, changes in epigenetic patterns are thought to play a crucial role in biological aging. Of the various aging-related epigenetic marks, DNA methylation has been the most extensively studied. A cross-sectional study involving 298 adults found that higher dietary intake of n-3 PUFAs was significantly associated with lower levels of ABCA1 DNA methylation [18]. Cognitive decline and frailty are widely recognized as common aspects of aging. In an 8.5-year follow-up of the Italian Longitudinal Study, higher PUFA intakes was associated with a lower risk of age-related cognitive decline [19]. Additionally, a Japanese longitudinal study found that older people with frailty components, such as skeletal muscle catabolism and physical dysfunction had lower serum levels of PUFAs, especially EPA and DHA [20]. This finding suggested that PUFAs may help mitigate frailty-related symptoms. However, a contrasting study by Doyeon et al. reported an inverse association between frailty and lower erythrocyte levels of long-chain n-3 PUFAs [21], indicating that further research is needed. Consistent with previous research, our findings showed that serum level of total n-3 PUFAs and its subtype including ALA, EPA and DHA were significant higher in non-accelerated aging group compared to accelerated aging group. Regression analyses indicated that higher levels of PUFAS, particularly n-3 PUFAs, were associated with delayed biological aging and exerted an anti-aging effect. Among these, DHA was identified as the strongest contributor in the mixed-effects analysis. Furthermore, sensitivity analyses indicated that higher levels of DHA were associated with a lower risk of all-cause mortality.

The aging process is believed to result from a combination of factors, including inflammation, oxidative stress, metabolic disorder and mitochondrial dysfunction. Among these, inflammation is considered particularly pivotal. Several studies have shown that PUFAs are involved in regulating inflammation process by producing different types of eicosanoids, inducing leukocyte chemotaxis and generating inflammatory cytokine interleukin-6 (IL-6), high-sensitivity C-reactive protein (hs-CRP), matrix metalloproteinase-2 (MMP-2), and matrix metalloproteinase-9 (MMP-9) [22, 23]. These inflammatory processes are thought to contribute significantly in the development of premature aging and age-related diseases. Moreover, eicosanoids derived from n-3 and n-6 PUFAs exhibit opposing pro-inflammatory and anti-inflammatory activities [24], which may elucidate their divergent roles in human health. Notably, our study found that the association between some n-6 PUFA subtypes and PhenoAge acceleration appears to be paradoxical. In the weighted multivariate regression analysis, GLA and AA showed no significant association with PhenoAge acceleration in Models 1 and 2, but were negatively correlated in Model 3. Conversely, DTA and n-6 DPA were significantly positively associated with PhenoAge acceleration in Models 1 and 2, but this association disappeared in Model 3 after adjusting for additional confounders. Subgroup analyses consistently indicated that age, sex, and race/ethnicity did not significantly modify these associations. The dual pro-inflammatory and anti-inflammatory effects of these fatty acids, which may vary depending on biological contexts and comorbidity, could help explain these observed discrepancies. Further studies are needed to clarify the underlying mechanisms and confirm these findings across different populations and settings.

Furthermore, this study discovered that the SII significantly mediated the relationships between AA, ALA, SDA, EPA, n-3 DPA, DHA and PhenoAge acceleration. This suggested that changes in SII may represent an important biological mechanism explaining the influence of PUFAs on PhenoAge acceleration. LA is an essential n-6 PUFA primarily obtained from plant sources such as nuts and seeds and acts as the precursor to both GLA and AA. The metabolic conversion of LA begins with its conversion to GLA via desaturase enzymes, followed by elongation to form dihomo-GLA (DGLA) through the action of elongase enzymes. Subsequently, DGLA undergoes further elongation and desaturation to be converted into AA [25]. Previous studies have suggested that LA and AA may exert anti-inflammatory effects. Thies et al. demonstrated that higher dietary intake of LA and AA did not lead to an increase in inflammatory cells count or inflammatory response in healthy individuals [26]. Furthermore, several studies have reported that moderate supplementation of LA can reduced the risks of cardiovascular disease [27], ischemic stroke [28], and enhance cognitive function [29]. The observed association between LA and a reduced risk of cardiovascular disease may also be attributed to its role in regulating lipid metabolism, which includes lowering concentrations of total cholesterol and low-density lipoprotein cholesterol in the blood [30]. Additionally, Milton Pereira et al. found that AA inhibited NLRP3 inflammasome activity in both human and mouse macrophages, which in turn reduced the production of pro-inflammatory cytokines IL-1β and IL-18 [31]. Our results align with these findings, showing that LA, GLA, and AA exhibit anti-aging effects and LA reduce the risk of CVD-related mortality (Supplementary Table 4). Mediation analysis indicated that the effect of AA on aging is partially mediated through its anti-inflammatory action. However, another study revealed that AA, as the precursor of inflammatory eicosanoids such as leukotriene B4 (LTB4) and prostaglandin E2 (PGE2), play a controversial role in inflammation [32]. LTB4 enhances endothelial permeability, triggers the release of lysosomal enzymes, and boosts reactive oxygen species production, which subsequently leads to the production of inflammatory cytokines such as IL-1, IL-6 and tumor necrosis factor-alpha (TNF-α). In contrast, PGE2 exhibits both pro-inflammatory and anti-inflammatory effects, a paradox attributed to its binding to different E prostanoid receptors [33]. Given these opposing roles, we hypothesized that the dual role of AA in inflammation may be influenced by various factors, potentially regulated by interactions with other PUFAs. It have been shown that n-3 PUFAs, such as EPA and DHA, inhibit the AA pathways leading to the production of inflammatory eicosanoids [34] by producing prostaglandin E(3) and leukotriene B(5). In addition, EPA and DHA exert anti-inflammatory effects by modifying cell membrane fatty acid composition, disrupting lipid rafts, inhibiting the pro-inflammatory transcription factor NF-κB, and activating the anti-inflammatory transcription factor PPAR-γ [35]. Therefore, we concluded that the anti-inflammatory and pro-inflammatory effects of AA may be context-dependent, influenced by disease backgrounds, though further research is needed to fully understand these complex mechanisms.

On the other hand, the combined effects of PUFAs on PhenoAge acceleration were assessed using the WQS and QGC models. The overall mixture of PUFAs showed a negative association with PhenoAge acceleration, indicating that higher levels of PUFAs were linked to slower aging. Among the PUFAs, DHA emerged as the most significant negative contributor, while DTA was identified as the primary positive contributor. Notably, DTA is converted into n-6 DPA through the action of the enzyme Δ6-desaturase, and n-6 DPA can then be further elongated to form DHA [36]. In our analysis, we found that both DTA and n-6 DPA were positively associated with accelerated aging in Models 1 and 2. Interestingly, low levels of DTA and n-6 DPA combined with high levels of EPA and DHA form what is known as the “high marine fatty acids” pattern. This pattern has been associated with better health in older adults, including reduced risks of depression, cognitive decline, and cardiovascular and cerebrovascular diseases [37, 38]. Also, there is interconversion between n-6 PUFAs and n-3 PUFAs but also antagonism due to the competition of converting enzymes. While our study did not further confirm that the higher n-6 to n-3 ratio was associated with an increasing PhenoAge acceleration in model 3, previous research has shown that maintaining a balance between n-3 and n-6 PUFAs is important for health. Currently, the ideal n-6 to n-3 ratio is between 1:1 and 4:1, but in most populations, this ratio is much higher, often ranging from 10:1 to 20:1 [39]. An imbalance in this ratio has been linked to several age-related diseases. Chang et al. found that higher plasma n-6 to n-3 PUFA ratio may be associated with shorter telomere length and a higher risk of CVD in the Chinese population [40]. Similarly, a randomized controlled trial by Calder et al. demonstrated that each one-unit decrease in the n-6 to n-3 PUFA ratio was linked to an estimated increase of 20 base pairs in telomere length [41]. Additionally, several previous studies have shown that an increased n-6 to n-3 ratio is associated with a higher risk of chronic disease [42], cognitive function impairment [43], and depression. The potential mechanism behind this association can be explained by the replacement of n-6 PUFAs with n-3 PUFAs, which related to anti-inflammatory activities, in cell membrane phospholipids [35]. Meanwhile, the shift in the n-6 PUFAs to n-3 PUFAs ratio in cell membranes has been shown to induce changes in various biological processes related to age-related decline, including the expression of pro- and anti-inflammatory lipid mediators and cytokines [35] and gene expression. However, further research is needed to clarify the optimal ratio of n-6 to n-3 PUFAs for maintaining health.

Based on current evidence, this is the first study to assess the relationship between serum PUFAs levels and biological aging acceleration as well as the mediating role of inflammation. There were following advantages of our study: Firstly, the PhenoAge algorithm was employed to determine biological age through readily detectable clinical indicators. This method offers a distinct advantage over traditional aging markers, such as telomere length and DNA methylation, by providing greater practicality for large-scale and population-wide applications. Secondly, compared to previous studies, we conducted a comprehensive assessment of both the individual and overall effects of PUFAs on biological aging acceleration. By dissecting the effect of each specific PUFA, the findings provided valuable insights into their respective contributions to aging processes. Thirdly, data from NHANES, used for analyses, provided a nationally representative sample of US adults. Various potential confounding factors were considered in this study as well to ensure the robustness of our findings. However, there are some limitations should be noted. Firstly, due to the cross-sectional design of our study, we cannot establish causality between serum PUFA and PhenoAge acceleration. Secondly, while we controlled for numerous potential confounders, the possibility of residual confounding cannot be fully excluded. Thirdly, serum PUFA data were collected during the 2011–2012 and 2013–2014 cycles, which may not accurately reflect the status among US adults due to changes in lifestyle and diet over the past decade. Longitudinal, large-scale population studies are needed to further elucidate the association between serum PUFA levels and accelerated biological aging.

## Conclusion

In conclusion, this study is the first to examine the association between serum omega-3 and omega-6 PUFAs and PhenoAge acceleration. We found that both total n-6 and n-3 PUFAs, along with specific subtypes (LA, GLA, AA, ALA, SDA, EPA, n-3 DPA, DHA), were negatively correlated with PhenoAge acceleration. These associations were consistent across subgroups, and non-linear relationships were observed for total n-6 PUFAs, LA, and ALA. Notably, DHA was identified as the main contributor to the negative association with PhenoAge acceleration. Mediation analysis showed that the SII mediated these relationships, suggesting that PUFAs may play an anti-inflammatory role in slowing PhenoAge acceleration. The findings of this study may help identify new targets for understanding the pathophysiology of aging and provide key insights for developing nutrition-based strategies aimed at anti-aging, with a focus on PUFAs.

## Supplementary Information

Below is the link to the electronic supplementary material.

Supplementary file 1 (DOCX 910 kb)

## Data Availability

No datasets were generated or analysed during the current study.
