# Supplementary material for: Systemic immune inflammation mediates the association of serum omega-3 and omega-6 polyunsaturated fatty acids with biological aging: a national population-based study
Source: Aging Clin Exp Res. 2025 Mar 8;37(1):74. doi: 10.1007/s40520-025-02964-2 (PMC11890405; doi:10.1007/s40520-025-02964-2)
Supplement: Supplementary file 1 — (DOCX 910 kb) [file 40520_2025_2964_MOESM1_ESM.docx]

***Supplementary Material***

1. **Supplementary Figures and Tables**

**1.1 Supplementary Figures**


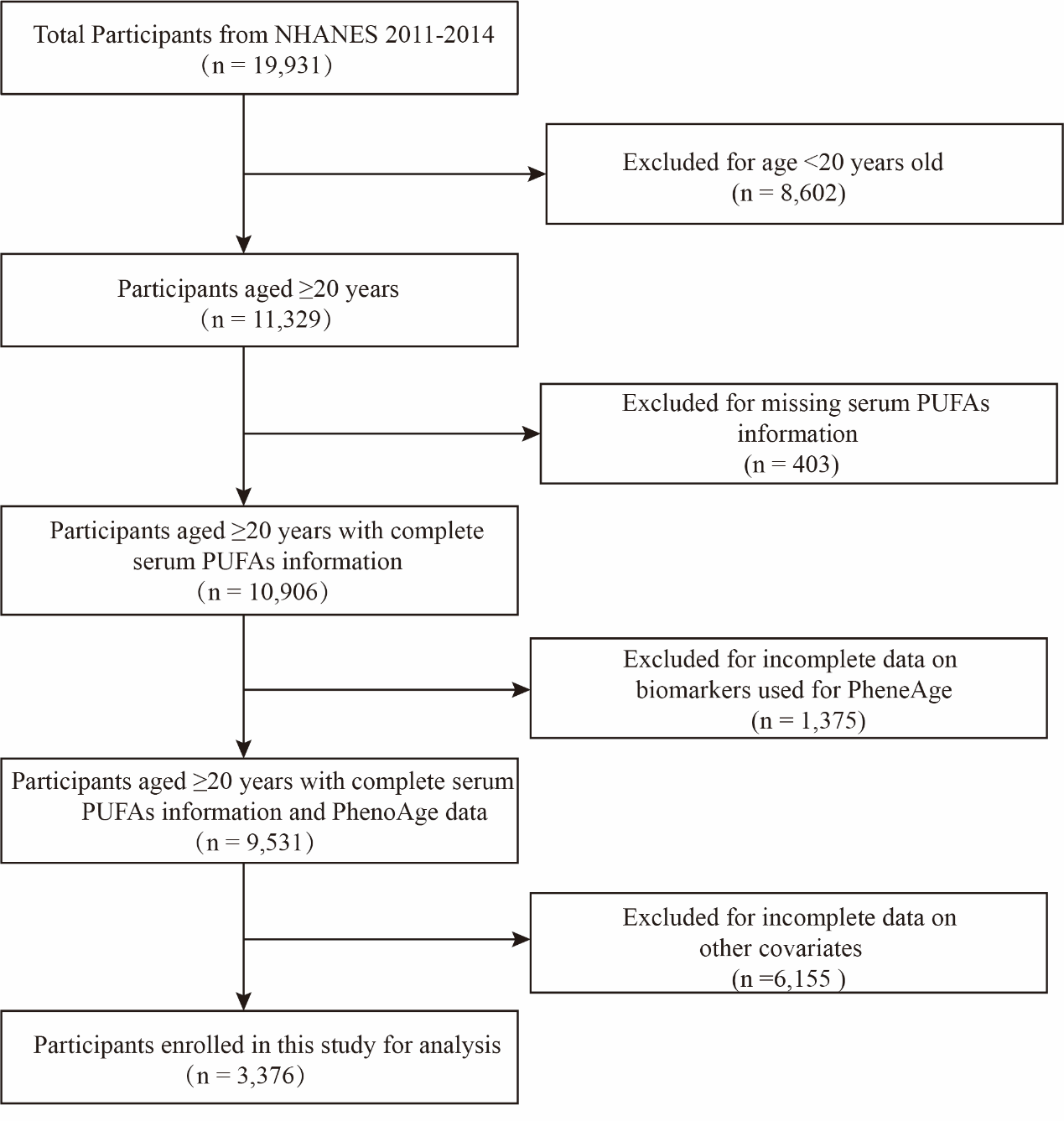


**Supplementary Figure 1:** Flow chart of participants selection. PUFAs, polyunsaturated fatty acids.


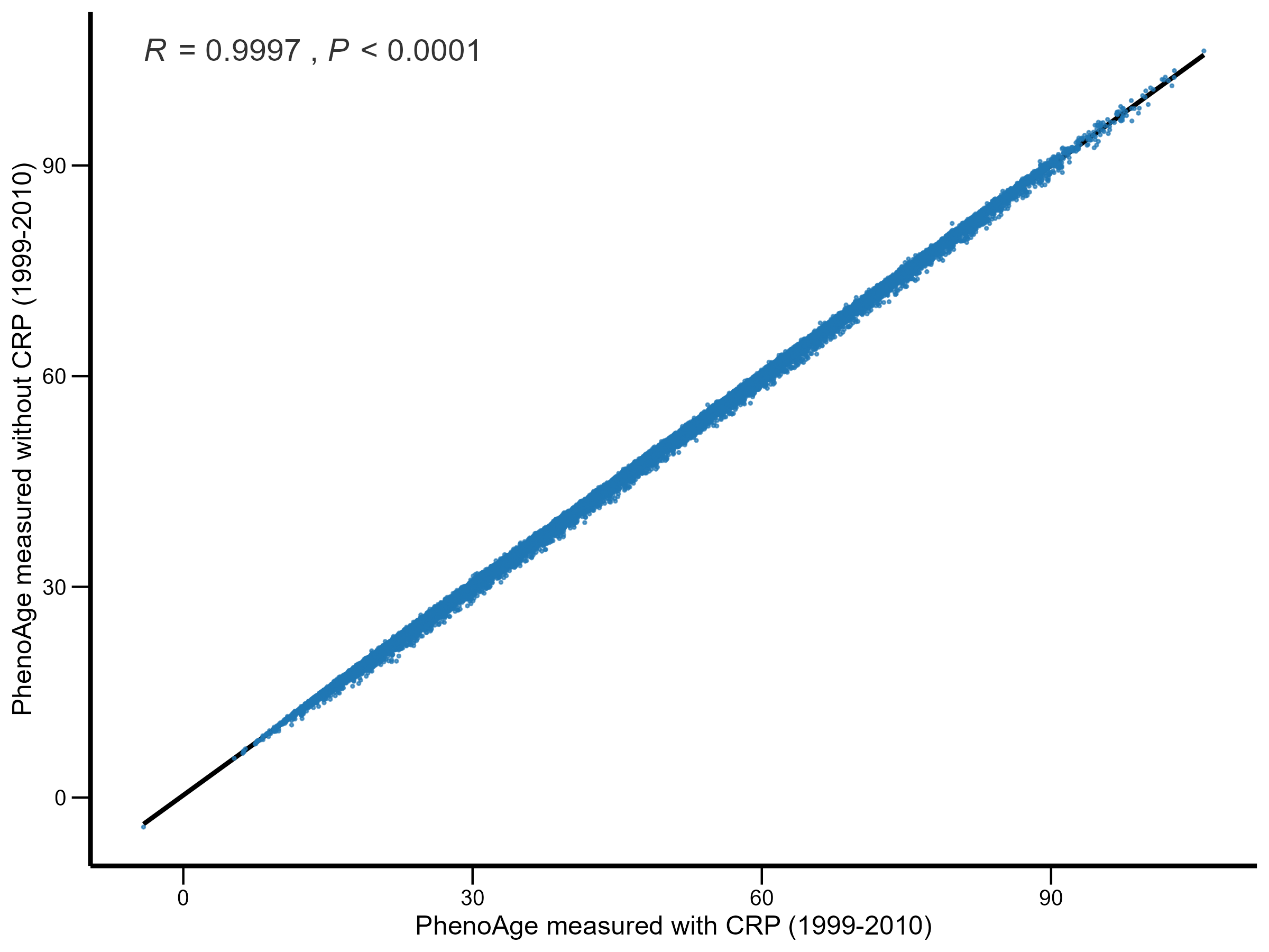


**Supplementary Figure 2:** Spearman correlation between PhenoAge measurements with and without CRP in the 1999-2010 NHANES data; CRP: C-reactive protein


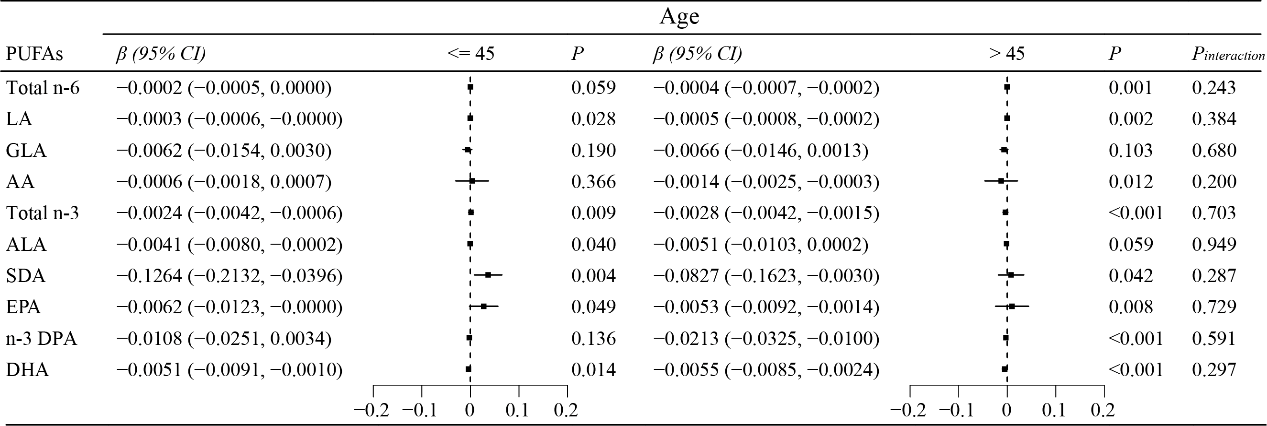


**Supplementary Figure 3.** Subgroup analysis for the association between serum omega-6 and omega-3 PUFAs and PhenoAge acceleration stratified by age.


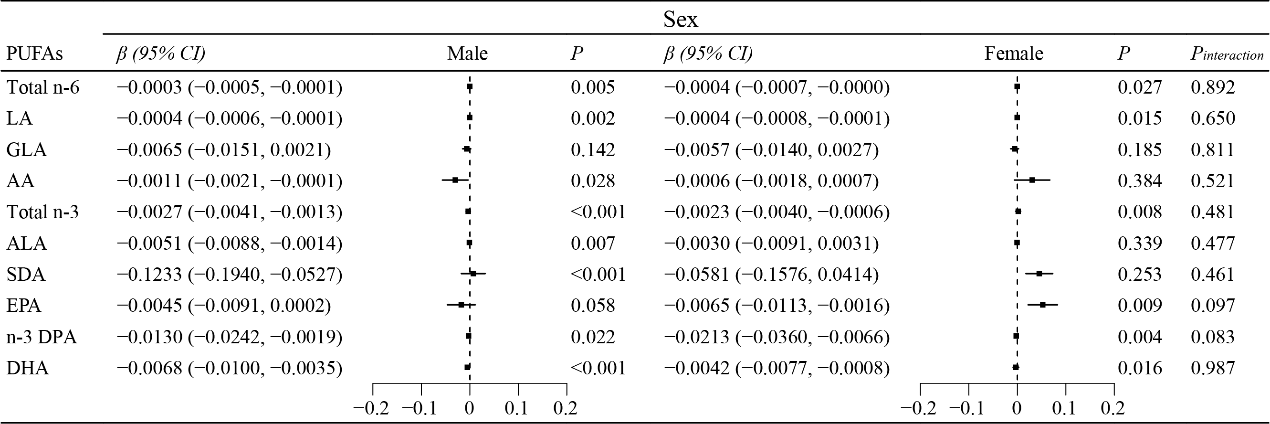


**Supplementary Figure 4.** Subgroup analysis for the association between serum omega-6 and omega-3 PUFAs and PhenoAge acceleration stratified by sex.


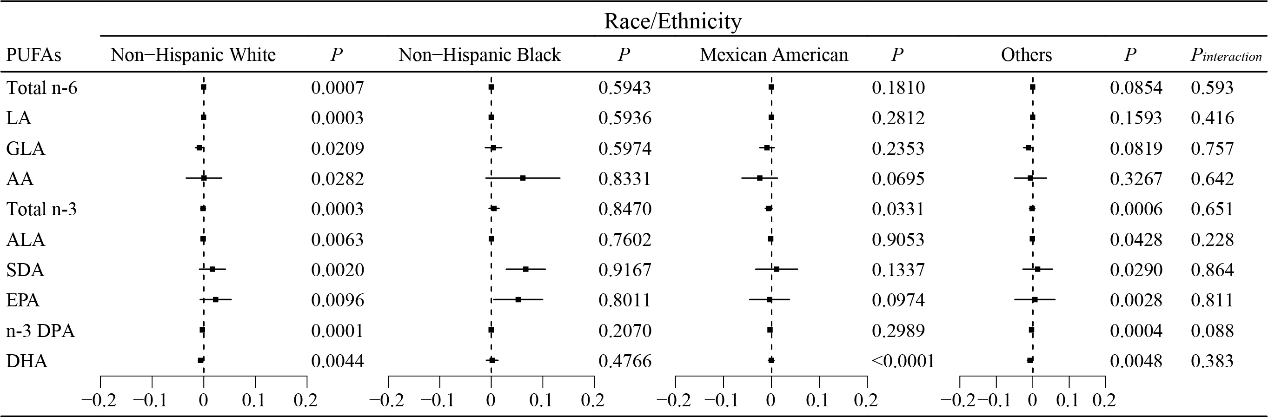


**Supplementary Figure 5.** Subgroup analysis for the association between serum omega-6 and omega-3 PUFAs and PhenoAge acceleration stratified by race/ethnicity

- 1. **Supplementary Tables**

**Supplementary Table 1.** Unweighted multivariate linear regression analysis of serum n-6 and n-3 PUFAs with PhenoAgeAccel.

|  | **Model 1** | | **Model 2** | | **Model 3** | |  |
| --- | --- | --- | --- | --- | --- | --- | --- |
|  | ***β (95% CI)*** | ***p*** | ***β (95% CI)*** | ***p*** | ***β (95% CI)*** | ***p*** |  |
| Total n-6 (continuous) | -0.0003 (-0.0005--0.0002) | <0.001 | -0.0003 (-0.0004--0.0001) | <0.001 | -0.0003 (-0.0004--0.0001) | <0.001 |  |
| Total n-6 (per SD) | -0.3706 (-0.5547--0.1865) | <0.001 | -0.3106 (-0.4894--0.1318) | <0.001 | -0.3052 (-0.4687--0.1417) | <0.001 |  |
| Tertile 1 | ref |  | ref |  | ref |  |  |
| Tertile 2 | -1.0295 (-1.4794--0.5797) | <0.001 | -0.9931 (-1.4258--0.5603) | <0.001 | -0.7621 (-1.1552--0.3691) | <0.001 |  |
| Tertile 3 | -1.1523 (-1.6022--0.7024) | <0.001 | -1.0281 (-1.4644--0.5919) | <0.001 | -0.9281 (-1.3269--0.5292) | <0.001 |  |
| *p* for trend |  | <0.001 |  | <0.001 |  | <0.001 |  |
| LA (continuous) | -0.0006 (-0.0007--0.0004) | <0.001 | -0.0004 (-0.0006--0.0002) | <0.001 | -0.0003 (-0.0005--0.0001) | <0.001 |  |
| LA (per SD) | -0.5402 (-0.7238--0.3566) | <0.001 | -0.3761 (-0.5565--0.1957) | <0.001 | -0.2999 (-0.4649--0.1350) | <0.001 |  |
| Tertile 1 | ref |  | ref |  | ref |  |  |
| Tertile 2 | -1.0765 (-1.5253--0.6277) | <0.001 | -0.8719 (-1.3052--0.4386) | <0.001 | -0.4461 (-0.8405--0.0516) | 0.027 |  |
| Tertile 3 | -1.3932 (-1.8404--0.9461) | <0.001 | -0.9858 (-1.4237--0.5479) | <0.001 | -0.7144 (-1.1148--0.3139) | <0.001 |  |
| *p* for trend |  | <0.001 |  | <0.001 |  | <0.001 |  |
| GLA (continuous) | 0.0087 (0.0030-0.0144) | 0.003 | 0.0036 (-0.0019-0.0092) | 0.203 | -0.0059 (-0.0110--0.0007) | 0.025 |  |
| GLA (per SD) | 0.2807 (0.0965-0.4650) | 0.003 | 0.1171 (-0.0630-0.2972) | 0.203 | -0.1904 (-0.3571--0.0237) | 0.025 |  |
| Tertile 1 | ref |  | ref |  | ref |  |  |
| Tertile 2 | 0.3165 (-0.1339-0.7670) | 0.168 | -0.2133 (-0.6519-0.2253) | 0.340 | -0.4540 (-0.8534--0.0547) | 0.026 |  |
| Tertile 3 | 0.8672 (0.4161-1.3182) | <0.001 | 0.3683 (-0.0738-0.8105) | 0.103 | -0.3547 (-0.7648-0.0554) | 0.090 |  |
| *p* for trend |  | <0.001 |  | 0.055 |  | 0.149 |  |
| EDA (continuous) | -0.0059 (-0.0266-0.0148) | 0.577 | 0.0095 (-0.0111-0.0300) | 0.366 | -0.0005 (-0.0192-0.0182) | 0.959 |  |
| EDA (per SD) | -0.0525 (-0.2370-0.1320) | 0.577 | 0.0844 (-0.0985-0.2674) | 0.366 | -0.0044 (-0.1707-0.1620) | 0.959 |  |
| Tertile 1 | ref |  | ref |  | ref |  |  |
| Tertile 2 | -0.4231 (-0.8748-0.0287) | 0.066 | -0.4313 (-0.8702-0.0077) | 0.054 | -0.5417 (-0.9396--0.1438) | 0.008 |  |
| Tertile 3 | -0.2923 (-0.7421-0.1575) | 0.203 | -0.0060 (-0.4533-0.4413) | 0.979 | -0.2284 (-0.6354-0.1786) | 0.271 |  |
| *p* for trend |  | 0.266 |  | 0.812 |  | 0.426 |  |
| HGLA (continuous) | 0.0000 (-0.0030-0.0031) | 0.986 | 0.0011 (-0.0020-0.0041) | 0.501 | -0.0021 (-0.0049-0.0008) | 0.151 |  |
| HGLA (per SD) | 0.0017 (-0.1828-0.1862) | 0.986 | 0.0632 (-0.1209-0.2472) | 0.501 | -0.1255 (-0.2967-0.0458) | 0.151 |  |
| Tertile 1 | ref |  | ref |  | ref |  |  |
| Tertile 2 | 0.0046 (-0.4459-0.4551) | 0.984 | -0.0168 (-0.4532-0.4195) | 0.940 | -0.1615 (-0.5590-0.2360) | 0.426 |  |
| Tertile 3 | -0.0686 (-0.5203-0.3830) | 0.766 | 0.0377 (-0.4113-0.4868) | 0.869 | -0.4216 (-0.8384--0.0048) | 0.047 |  |
| *p* for trend |  | 0.755 |  | 0.858 |  | 0.046 |  |
| AA (continuous) | 0.0012 (0.0004-0.0019) | 0.002 | -0.0003 (-0.0010-0.0004) | 0.408 | -0.0009 (-0.0016--0.0003) | 0.006 |  |
| AA (per SD) | 0.2971 (0.1129-0.4814) | 0.002 | -0.0780 (-0.2628-0.1067) | 0.408 | -0.2378 (-0.4060--0.0696) | 0.006 |  |
| Tertile 1 | ref |  | ref |  | ref |  |  |
| Tertile 2 | 0.4480 (-0.0031-0.8991) | 0.052 | -0.1080 (-0.5474-0.3314) | 0.630 | -0.2306 (-0.6282-0.1671) | 0.256 |  |
| Tertile 3 | 0.7057 (0.2544-1.1570) | 0.002 | -0.2240 (-0.6761-0.2282) | 0.331 | -0.5331 (-0.9440--0.1222) | 0.011 |  |
| *p* for trend |  | 0.002 |  | 0.332 |  | 0.011 |  |
| DTA (continuous) | 0.0853 (0.0688-0.1019) | <0.001 | 0.0647 (0.0483-0.0812) | <0.001 | 0.0281 (0.0126-0.0436) | <0.001 |  |
| DTA (per SD) | 0.9367 (0.7549-1.1185) | <0.001 | 0.7106 (0.5305-0.8907) | <0.001 | 0.3088 (0.1388-0.4788) | <0.001 |  |
| Tertile 1 | ref |  | ref |  | ref |  |  |
| Tertile 2 | 1.2897 (0.8440-1.7354) | <0.001 | 0.7930 (0.3536-1.2324) | <0.001 | 0.2810 (-0.1219-0.6840) | 0.172 |  |
| Tertile 3 | 2.1364 (1.6904-2.5824) | <0.001 | 1.5557 (1.1126-1.9988) | <0.001 | 0.6623 (0.2466-1.0780) | 0.002 |  |
| *p* for trend |  | <0.001 |  | <0.001 |  | 0.002 |  |
| DPA (continuous) | 0.0619 (0.0420-0.0818) | <0.001 | 0.0524 (0.0329-0.0719) | <0.001 | 0.0243 (0.0063-0.0424) | 0.008 |  |
| DPA (per SD) | 0.5703 (0.3868-0.7538) | <0.001 | 0.4825 (0.3031-0.6619) | <0.001 | 0.2242 (0.0582-0.3902) | 0.008 |  |
| Tertile 1 | ref |  | ref |  | ref |  |  |
| Tertile 2 | 0.8030 (0.3544-1.2516) | <0.001 | 0.5960 (0.1618-1.0302) | 0.007 | 0.2578 (-0.1383-0.6539) | 0.202 |  |
| Tertile 3 | 1.1289 (0.6778-1.5800) | <0.001 | 0.8405 (0.3981-1.2830) | <0.001 | 0.2230 (-0.1855-0.6314) | 0.285 |  |
| *p* for trend |  | <0.001 |  | <0.001 |  | 0.318 |  |
| Total n-3 (continuous) | | -0.0035 (-0.0045--0.0025) | <0.001 | -0.0034 (-0.0044--0.0024) | <0.001 | -0.0025 (-0.0034--0.0015) | <0.001 |
| Total n-3 (per SD) | | -0.6392 (-0.8224--0.4559) | <0.001 | -0.6143 (-0.8026--0.4261) | <0.001 | -0.4448 (-0.6172--0.2725) | <0.001 |
| Tertile 1 | | ref |  | ref |  | ref |  |
| Tertile 2 | | -0.2372 (-0.6860-0.2115) | 0.300 | -0.4041 (-0.8419-0.0337) | 0.070 | -0.2881 (-0.6878-0.1116) | 0.158 |
| Tertile 3 | | -1.4742 (-1.9229--1.0254) | <0.001 | -1.5385 (-1.9961--1.0809) | <0.001 | -1.0672 (-1.4898--0.6445) | <0.001 |
| *p* for trend | |  | <0.001 |  | <0.001 |  | <0.001 |
| ALA (continuous) | | -0.0037 (-0.0070--0.0004) | 0.026 | -0.0013 (-0.0045-0.0019) | 0.420 | -0.0029 (-0.0059--0.0000) | 0.049 |
| ALA (per SD) | | -0.2087 (-0.3931--0.0244) | 0.026 | -0.0744 (-0.2554-0.1066) | 0.420 | -0.1654 (-0.3304--0.0004) | 0.049 |
| Tertile 1 | | ref |  | ref |  | ref |  |
| Tertile 2 | | -0.9277 (-1.3779--0.4775) | <0.001 | -0.7455 (-1.1814--0.3097) | <0.001 | -0.6582 (-1.0536--0.2627) | 0.001 |
| Tertile 3 | | -1.0297 (-1.4798--0.5797) | <0.001 | -0.6980 (-1.1434--0.2525) | 0.002 | -0.8480 (-1.2539--0.4421) | <0.001 |
| *p* for trend | |  | <0.001 |  | 0.008 |  | <0.001 |
| SDA (continuous) | | -0.0184 (-0.0747-0.0379) | 0.522 | -0.0275 (-0.0824-0.0273) | 0.325 | -0.0787 (-0.1287--0.0287) | 0.002 |
| SDA (per SD) | | -0.0603 (-0.2448-0.1242) | 0.522 | -0.0902 (-0.2698-0.0894) | 0.325 | -0.2579 (-0.4218--0.0941) | 0.002 |
| Tertile 1 | | ref |  | ref |  | ref |  |
| Tertile 2 | | 0.0906 (-0.3609-0.5422) | 0.694 | -0.2320 (-0.6697-0.2057) | 0.299 | -0.5235 (-0.9207--0.1263) | 0.010 |
| Tertile 3 | | 0.1520 (-0.2996-0.6036) | 0.509 | -0.0910 (-0.5334-0.3515) | 0.687 | -0.6100 (-1.0149--0.2050) | 0.003 |
| *p* for trend | |  | 0.527 |  | 0.844 |  | 0.009 |
| EPA (continuous) | | -0.0086 (-0.0116--0.0055) | <0.001 | -0.0089 (-0.0120--0.0058) | <0.001 | -0.0058 (-0.0086--0.0030) | <0.001 |
| EPA (per SD) | | -0.5149 (-0.6986--0.3313) | <0.001 | -0.5327 (-0.7182--0.3471) | <0.001 | -0.3472 (-0.5168--0.1775) | <0.001 |
| Tertile 1 | | ref |  | ref |  | ref |  |
| Tertile 2 | | -0.2438 (-0.6936-0.2061) | 0.288 | -0.5994 (-1.0377--0.1611) | 0.007 | -0.5466 (-0.9463--0.1469) | 0.007 |
| Tertile 3 | | -1.0376 (-1.4887--0.5866) | <0.001 | -1.2697 (-1.7256--0.8138) | <0.001 | -0.9608 (-1.3792--0.5424) | <0.001 |
| *p* for trend | |  | <0.001 |  | <0.001 |  | <0.001 |
| DPA (continuous) | | -0.0089 (-0.0179-0.0002) | 0.054 | -0.0157 (-0.0247--0.0067) | <0.001 | -0.0174 (-0.0255--0.0092) | <0.001 |
| DPA (per SD) | | -0.1813 (-0.3657-0.0031) | 0.054 | -0.3214 (-0.5051--0.1377) | <0.001 | -0.3545 (-0.5211--0.1880) | <0.001 |
| Tertile 1 | | ref |  | ref |  | ref |  |
| Tertile 2 | | -0.0070 (-0.4585-0.4445) | 0.976 | -0.3203 (-0.7581-0.1176) | 0.152 | -0.3165 (-0.7132-0.0802) | 0.118 |
| Tertile 3 | | -0.3640 (-0.8159-0.0879) | 0.114 | -0.7361 (-1.1849--0.2872) | 0.001 | -0.7448 (-1.1526--0.3371) | <0.001 |
| *p* for trend | |  | 0.096 |  | 0.001 |  | <0.001 |
| DHA (continuous) | | -0.0090 (-0.0111--0.0069) | <0.001 | -0.0091 (-0.0113--0.0069) | <0.001 | -0.0056 (-0.0077--0.0036) | <0.001 |
| DHA (per SD) | | -0.7894 (-0.9720--0.6068) | <0.001 | -0.7941 (-0.9861--0.6021) | <0.001 | -0.4924 (-0.6712--0.3136) | <0.001 |
| Tertile 1 | | ref |  | ref |  | ref |  |
| Tertile 2 | | -0.6135 (-1.0605--0.1666) | 0.007 | -0.8015 (-1.2364--0.3666) | <0.001 | -0.6949 (-1.0946--0.2951) | <0.001 |
| Tertile 3 | | -1.7208 (-2.1696--1.2720) | <0.001 | -1.8736 (-2.3331--1.4142) | <0.001 | -1.1909 (-1.6222--0.7597) | <0.001 |
| *p* for trend | |  | <0.001 |  | <0.001 |  | <0.001 |
| n-6/n-3 (continuous) | | 0.1041 (0.0586-0.1496) | <0.001 | 0.1183 (0.0708-0.1657) | <0.001 | 0.0765 (0.0323-0.1206) | <0.001 |
| n-6/n-3 (per SD) | | 0.4207 (0.2367-0.6046) | <0.001 | 0.4780 (0.2862-0.6697) | <0.001 | 0.3090 (0.1306-0.4875) | <0.001 |
| Tertile 1 | | ref |  | ref |  | ref |  |
| Tertile 2 | | 1.1793 (0.7295-1.6291) | <0.001 | 1.1122 (0.6688-1.5556) | <0.001 | 0.6711 (0.2628-1.0794) | 0.001 |
| Tertile 3 | | 1.0523 (0.6025-1.5021) | <0.001 | 1.1759 (0.7143-1.6375) | <0.001 | 0.7576 (0.3281-1.1871) | <0.001 |
| *p* for trend | |  | <0.001 |  | <0.001 |  | <0.001 |

Model 1: Unadjusted.

Model 2: Adjusted for age, sex, and race/ethnicity.

Model 3: Adjusted for age, sex, race/ethnicity, education, poverty income ratio, body mass index, smoking status, diabetes, hypertension, cardiovascular disease, cancer.

CI: Confidence interval; ref: reference level; PhenoAgeAceel: phenotypic age acceleration.

**Supplementary Table 2.** Weighted multivariate logistic regression analysis of serum n-6 and n-3 PUFAs with accelerated aging.

|  | **Model 1** | | **Model 2** | | **Model 3** | |  |
| --- | --- | --- | --- | --- | --- | --- | --- |
|  | ***OR (95% CI)*** | ***p*** | ***OR (95% CI)*** | ***p*** | ***OR (95% CI)*** | ***p*** |  |
| Total n-6 (continuous) | 0.9999 (0.9998, 1.0000) | 0.162 | 0.9999 (0.9998, 1.0000) | 0.197 | 0.9999 (0.9997, 1.0000) | 0.127 |  |
| Quartile 1 | ref |  | ref |  | ref |  |  |
| Quartile 2 | 0.6756 (0.4822, 0.9465) | 0.024 | 0.7020 (0.4908, 1.0042) | 0.053 | 0.7042 (0.4848, 1.0228) | 0.063 |  |
| Quartile 3 | 0.6056 (0.4347, 0.8438) | 0.004 | 0.5944 (0.4267, 0.8278) | 0.003 | 0.6158 (0.4197, 0.9035) | 0.017 |  |
| Quartile 4 | 0.7382 (0.5408, 1.0077) | 0.056 | 0.7421 (0.5387, 1.0224) | 0.067 | 0.6921 (0.4783, 1.0016) | 0.051 |  |
| LA (continuous) | 0.9998 (0.9997, 1.0000) | 0.037 | 0.9999 (0.9997, 1.0000) | 0.081 | 0.9999 (0.9997, 1.0000) | 0.098 |  |
| Quartile 1 | ref |  | ref |  | ref |  |  |
| Quartile 2 | 0.6294 (0.4530, 0.8746) | 0.007 | 0.6846 (0.4864, 0.9636) | 0.031 | 0.7511 (0.4975, 1.1338) | 0.156 |  |
| Quartile 3 | 0.5419 (0.3601, 0.8156) | 0.005 | 0.5656 (0.3715, 0.8611) | 0.010 | 0.6013 (0.3803, 0.9507) | 0.032 |  |
| Quartile 4 | 0.5858 (0.4240, 0.8095) | 0.002 | 0.6247 (0.4463, 0.8745) | 0.008 | 0.6416 (0.4440, 0.9273) | 0.022 |  |
| GLA (continuous) | 1.0015 (0.9991, 1.0039) | 0.217 | 1.0009 (0.9984, 1.0034) | 0.470 | 0.9977 (0.9946, 1.0008) | 0.134 |  |
| Quartile 1 | ref |  | ref |  | ref |  |  |
| Quartile 2 | 1.0707 (0.8795, 1.3036) | 0.483 | 0.9799 (0.7868, 1.2204) | 0.850 | 0.8706 (0.6841, 1.1080) | 0.234 |  |
| Quartile 3 | 1.2386 (0.8920, 1.7197) | 0.193 | 1.1158 (0.7973, 1.5616) | 0.507 | 0.8716 (0.6563, 1.1575) | 0.312 |  |
| Quartile 4 | 1.2485 (0.9692, 1.6081) | 0.083 | 1.1529 (0.8778, 1.5142) | 0.292 | 0.8472 (0.6375, 1.1258) | 0.228 |  |
| EDA (continuous) | 1.0066 (0.9911, 1.0223) | 0.392 | 1.0091 (0.9925, 1.0260) | 0.273 | 1.0038 (0.9850, 1.0229) | 0.675 |  |
| Quartile 1 | ref |  | ref |  | ref |  |  |
| Quartile 2 | 0.8848 (0.6639, 1.1791) | 0.390 | 0.8832 (0.6628, 1.1770) | 0.381 | 0.8655 (0.6325, 1.1842) | 0.335 |  |
| Quartile 3 | 0.8586 (0.6426, 1.1471) | 0.291 | 0.8536 (0.6394, 1.1396) | 0.269 | 0.7861 (0.5645, 1.0948) | 0.139 |  |
| Quartile 4 | 1.2845 (0.8886, 1.8566) | 0.175 | 1.3436 (0.9140, 1.9751) | 0.127 | 1.2084 (0.7955, 1.8356) | 0.343 |  |
| HGLA (continuous) | 1.0009 (0.9990, 1.0027) | 0.358 | 1.0015 (0.9995, 1.0035) | 0.131 | 1.0002 (0.9980, 1.0024) | 0.845 |  |
| Quartile 1 | ref |  | ref |  | ref |  |  |
| Quartile 2 | 0.7757 (0.5815, 1.0349) | 0.082 | 0.7844 (0.5870, 1.0482) | 0.097 | 0.7983 (0.5878, 1.0843) | 0.135 |  |
| Quartile 3 | 0.8008 (0.6148, 1.0431) | 0.096 | 0.8509 (0.6526, 1.1095) | 0.221 | 0.8047 (0.5947, 1.0888) | 0.143 |  |
| Quartile 4 | 1.0386 (0.7620, 1.4155) | 0.804 | 1.1472 (0.8245, 1.5962) | 0.399 | 0.9636 (0.6603, 1.4064) | 0.835 |  |
| AA (continuous) | 1.0003 (0.9999, 1.0008) | 0.164 | 1.0000 (0.9995, 1.0005) | 0.876 | 0.9998 (0.9992, 1.0003) | 0.358 |  |
| Quartile 1 | ref |  | ref |  | ref |  |  |
| Quartile 2 | 1.1747 (0.8954, 1.5410) | 0.235 | 1.0831 (0.8174, 1.4353) | 0.564 | 1.0166 (0.7572, 1.3649) | 0.905 |  |
| Quartile 3 | 1.1115 (0.8715, 1.4177) | 0.381 | 0.9660 (0.7461, 1.2507) | 0.785 | 0.8659 (0.6199, 1.2097) | 0.367 |  |
| Quartile 4 | 1.2751 (0.9530, 1.7062) | 0.098 | 1.0567 (0.7758, 1.4392) | 0.716 | 0.8550 (0.6080, 1.2024) | 0.337 |  |
| DTA (continuous) | 1.0258 (1.0173, 1.0345) | <0.001 | 1.0243 (1.0156, 1.0332) | <0.001 | 1.0113 (1.0004, 1.0222) | 0.043 |  |
| Quartile 1 | ref |  | ref |  | ref |  |  |
| Quartile 2 | 1.1620 (0.8776, 1.5384) | 0.283 | 1.1434 (0.8576, 1.5245) | 0.346 | 0.9245 (0.6744, 1.2673) | 0.597 |  |
| Quartile 3 | 1.7666 (1.3817, 2.2587) | <0.001 | 1.6992 (1.3091, 2.2056) | <0.001 | 1.1688 (0.9277, 1.4726) | 0.167 |  |
| Quartile 4 | 2.1153 (1.5941, 2.8068) | <0.001 | 2.0046 (1.4925, 2.6926) | <0.001 | 1.3304 (0.9146, 1.9352) | 0.123 |  |
| n-6 DPA (continuous) | 1.0234 (1.0120, 1.0350) | <0.001 | 1.0243 (1.0122, 1.0365) | <0.001 | 1.0132 (0.9978, 1.0288) | 0.088 |  |
| Quartile 1 | ref |  | ref |  | ref |  |  |
| Quartile 2 | 1.2867 (0.9184, 1.8026) | 0.137 | 1.3167 (0.9403, 1.8437) | 0.105 | 1.1586 (0.8707, 1.5416) | 0.283 |  |
| Quartile 3 | 1.5652 (1.1874, 2.0632) | 0.002 | 1.5497 (1.1456, 2.0965) | 0.006 | 1.2914 (0.9705, 1.7185) | 0.075 |  |
| Quartile 4 | 1.7126 (1.2675, 2.3139) | 0.001 | 1.7277 (1.2554, 2.3777) | 0.002 | 1.3339 (0.9353, 1.9023) | 0.102 |  |
| Total n-3 (continuous) | | 0.9991 (0.9981, 1.0000) | 0.056 | 0.9988 (0.9977, 0.9998) | 0.026 | 0.9991 (0.9982, 0.9999) | **0.039** |
| Quartile 1 | | ref |  | ref |  | ref |  |
| Quartile 2 | | 0.9648 (0.7589, 1.2267) | 0.763 | 0.8961 (0.6858, 1.1709) | 0.406 | 0.9493 (0.7022, 1.2834) | 0.714 |
| Quartile 3 | | 0.8079 (0.6154, 1.0606) | 0.120 | 0.7181 (0.5438, 0.9482) | 0.022 | 0.7872 (0.5725, 1.0825) | 0.128 |
| Quartile 4 | | 0.6622 (0.4617, 0.9497) | 0.026 | 0.5729 (0.3840, 0.8547) | 0.008 | 0.6981 (0.4904, 0.9938) | **0.047** |
| ALA (continuous) | | 0.9994 (0.9972, 1.0016) | 0.578 | 0.9997 (0.9974, 1.0020) | 0.811 | 0.9989 (0.9963, 1.0016) | 0.404 |
| Quartile 1 | | ref |  | ref |  | ref |  |
| Quartile 2 | | 0.6716 (0.5116, 0.8816) | 0.006 | 0.6838 (0.5132, 0.9111) | 0.012 | 0.6989 (0.5186, 0.9421) | 0.023 |
| Quartile 3 | | 0.6777 (0.4807, 0.9554) | 0.028 | 0.6968 (0.4859, 0.9990) | 0.049 | 0.6500 (0.4231, 0.9984) | 0.049 |
| Quartile 4 | | 0.8716 (0.6222, 1.2211) | 0.411 | 0.9125 (0.6352, 1.3109) | 0.607 | 0.8371 (0.5812, 1.2057) | 0.309 |
| SDA (continuous) | | 0.9932 (0.9670, 1.0201) | 0.606 | 0.9904 (0.9617, 1.0200) | 0.508 | 0.9677 (0.9343, 1.0021) | 0.064 |
| Quartile 1 | | ref |  | ref |  | ref |  |
| Quartile 2 | | 1.0659 (0.7772, 1.4618) | 0.682 | 1.0128 (0.7252, 1.4146) | 0.938 | 0.9326 (0.6622, 1.3135) | 0.665 |
| Quartile 3 | | 1.1338 (0.8904, 1.4436) | 0.297 | 1.0497 (0.8133, 1.3548) | 0.698 | 0.9138 (0.6998, 1.1931) | 0.476 |
| Quartile 4 | | 1.0398 (0.7637, 1.4158) | 0.798 | 1.0009 (0.7192, 1.3930) | 0.996 | 0.8342 (0.6061, 1.1482) | 0.240 |
| EPA (continuous) | | 0.9972 (0.9945, 0.9999) | 0.043 | 0.9961 (0.9928, 0.9993) | 0.019 | 0.9972 (0.9946, 0.9998) | **0.037** |
| Quartile 1 | | ref |  | ref |  | ref |  |
| Quartile 2 | | 0.8698 (0.6735, 1.1233) | 0.274 | 0.7739 (0.5813, 1.0303) | 0.077 | 0.7728 (0.5538, 1.0785) | 0.118 |
| Quartile 3 | | 0.8580 (0.6647, 1.1074) | 0.230 | 0.7369 (0.5652, 0.9607) | 0.026 | 0.7884 (0.6056, 1.0264) | 0.073 |
| Quartile 4 | | 0.6239 (0.4602, 0.8459) | 0.004 | 0.5152 (0.3613, 0.7345) | <0.001 | 0.6214 (0.4492, 0.8597) | **0.008** |
| n-3 DPA (continuous) | | 0.9972 (0.9912, 1.0032) | 0.349 | 0.9949 (0.9880, 1.0018) | 0.142 | 0.9931 (0.9865, 0.9997) | **0.042** |
| Quartile 1 | | ref |  | ref |  | ref |  |
| Quartile 2 | | 1.0289 (0.7515, 1.4086) | 0.854 | 0.9453 (0.6906, 1.2940) | 0.715 | 0.8933 (0.6518, 1.2242) | 0.450 |
| Quartile 3 | | 1.1364 (0.8317, 1.5526) | 0.409 | 1.0115 (0.7236, 1.4140) | 0.944 | 0.9599 (0.7090, 1.2996) | 0.773 |
| Quartile 4 | | 0.9068 (0.6476, 1.2697) | 0.557 | 0.7982 (0.5566, 1.1448) | 0.209 | 0.7400 (0.5201, 1.0529) | 0.088 |
| DHA (continuous) | | 0.9977 (0.9956, 0.9998) | 0.034 | 0.9967 (0.9943, 0.9991) | 0.010 | 0.9981 (0.9962, 1.0000) | 0.051 |
| Quartile 1 | | ref |  | ref |  | ref |  |
| Quartile 2 | | 0.7556 (0.5394, 1.0587) | 0.100 | 0.6910 (0.4793, 0.9962) | 0.048 | 0.7731 (0.5136, 1.1638) | 0.196 |
| Quartile 3 | | 0.6280 (0.4479, 0.8806) | 0.009 | 0.5167 (0.3714, 0.7187) | <0.001 | 0.5843 (0.4133, 0.8260) | 0.005 |
| Quartile 4 | | 0.5697 (0.3836, 0.8461) | 0.007 | 0.4540 (0.2968, 0.6944) | <0.001 | 0.6561 (0.4529, 0.9505) | **0.029** |
| n-6/n-3 (continuous) | | 1.0231 (0.9938, 1.0533) | 0.119 | 1.0414 (1.0059, 1.0782) | 0.024 | 1.0184 (0.9879, 1.0499) | 0.219 |
| Quartile 1 | | ref |  | ref |  | ref |  |
| Quartile 2 | | 1.3475 (1.0137, 1.7913) | 0.041 | 1.3782 (1.0198, 1.8625) | 0.038 | 1.1574 (0.8073, 1.6594) | 0.394 |
| Quartile 3 | | 1.4395 (1.0690, 1.9383) | 0.018 | 1.6311 (1.1693, 2.2753) | 0.006 | 1.2784 (0.9616, 1.6996) | 0.085 |
| Quartile 4 | | 1.3067 (0.8988, 1.8998) | 0.155 | 1.5428 (1.0139, 2.3476) | 0.043 | 1.1981 (0.8245, 1.7411) | 0.313 |

Model 1: Unadjusted.

Model 2: Adjusted for age, sex, and race/ethnicity.

Model 3: Adjusted for age, sex, race/ethnicity, education, poverty income ratio, body mass index, smoking status, diabetes, hypertension, cardiovascular disease, cancer.

OR: Odds Ratio; CI: Confidence interval; ref: reference level; PhenoAgeAceel: phenotypic age acceleration.

**Supplementary Table 3. Association of serum n-6 and n-3 PUFAs with all-cause mortality (Weighted)**

|  | **Model 1** | | **Model 2** | | **Model 3** | |  |
| --- | --- | --- | --- | --- | --- | --- | --- |
|  | ***HR (95% CI)*** | ***p*** | ***HR (95% CI)*** | ***p*** | ***HR (95% CI)*** | ***p*** |  |
| Total n-6 (continuous) | 0.9998 (0.9996, 1.0000) | 0.022 | 0.9998 (0.9996, 1.0000) | 0.044 | 0.9998 (0.9996, 1.0000) | 0.087 |  |
| Quartile 1 | ref |  | ref |  | ref |  |  |
| Quartile 2 | 0.5912 (0.4151, 0.8419) | 0.004 | 0.6912 (0.4826, 0.9900) | 0.044 | 0.7190 (0.4828, 1.0707) | 0.104 |  |
| Quartile 3 | 0.6552 (0.4425, 0.9701) | 0.035 | 0.6744 (0.4536, 1.0026) | 0.052 | 0.7609 (0.5036, 1.1495) | 0.194 |  |
| Quartile 4 | 0.6323 (0.4273, 0.9355) | 0.022 | 0.6350 (0.4033, 1.0000) | 0.050 | 0.6963 (0.4442, 1.0916) | 0.115 |  |
| LA (continuous) | 0.9997 (0.9994, 0.9999) | 0.009 | 0.9998 (0.9995, 1.0000) | 0.034 | 0.9998 (0.9996, 1.0000) | 0.074 |  |
| Quartile 1 | ref |  | ref |  | ref |  |  |
| Quartile 2 | 0.5086 (0.3421, 0.7561) | <0.001 | 0.6677 (0.4551, 0.9797) | 0.039 | 0.7225 (0.4933, 1.0582) | 0.095 |  |
| Quartile 3 | 0.5544 (0.3953, 0.7775) | <0.001 | 0.7483 (0.5222, 1.0724) | 0.114 | 0.8854 (0.6468, 1.2121) | 0.448 |  |
| Quartile 4 | 0.4701 (0.2967, 0.7449) | 0.001 | 0.5191 (0.3159, 0.8530) | 0.010 | 0.5828 (0.3535, 0.9607) | 0.034 |  |
| GLA (continuous) | 1.0004 (0.9958, 1.0051) | 0.860 | 0.9997 (0.9943, 1.0052) | 0.923 | 0.9994 (0.9931, 1.0058) | 0.859 |  |
| Quartile 1 | ref |  | ref |  | ref |  |  |
| Quartile 2 | 1.2260 (0.8096, 1.8565) | 0.336 | 1.0416 (0.6541, 1.6586) | 0.864 | 1.0348 (0.6379, 1.6786) | 0.890 |  |
| Quartile 3 | 0.8191 (0.4670, 1.4366) | 0.486 | 0.6874 (0.3669, 1.2881) | 0.242 | 0.6583 (0.3602, 1.2031) | 0.174 |  |
| Quartile 4 | 1.1677 (0.7194, 1.8955) | 0.530 | 1.0120 (0.5932, 1.7264) | 0.965 | 1.0196 (0.5658, 1.8374) | 0.948 |  |
| EDA (continuous) | 1.0028 (0.9850, 1.0208) | 0.762 | 1.0022 (0.9778, 1.0271) | 0.863 | 0.9981 (0.9735, 1.0233) | 0.882 |  |
| Quartile 1 | ref |  | ref |  | ref |  |  |
| Quartile 2 | 1.1181 (0.7453, 1.6774) | 0.590 | 0.9058 (0.6408, 1.2804) | 0.575 | 0.9040 (0.6500, 1.2573) | 0.549 |  |
| Quartile 3 | 0.8874 (0.5399, 1.4586) | 0.637 | 0.6651 (0.4187, 1.0563) | 0.084 | 0.7002 (0.4580, 1.0705) | 0.100 |  |
| Quartile 4 | 1.0987 (0.7094, 1.7019) | 0.673 | 0.9068 (0.5820, 1.4129) | 0.666 | 0.8313 (0.5448, 1.2683) | 0.391 |  |
| HGLA (continuous) | 0.9993 (0.9963, 1.0024) | 0.662 | 1.0008 (0.9973, 1.0044) | 0.643 | 1.0012 (0.9973, 1.0051) | 0.538 |  |
| Quartile 1 | ref |  | ref |  | ref |  |  |
| Quartile 2 | 0.8120 (0.4902, 1.3451) | 0.419 | 0.8028 (0.4702, 1.3708) | 0.421 | 0.8141 (0.4756, 1.3935) | 0.453 |  |
| Quartile 3 | 0.8885 (0.5293, 1.4914) | 0.655 | 1.0673 (0.5937, 1.9185) | 0.828 | 1.1670 (0.6303, 2.1606) | 0.623 |  |
| Quartile 4 | 0.8220 (0.4888, 1.3821) | 0.460 | 1.0165 (0.5337, 1.9361) | 0.960 | 1.0796 (0.5670, 2.0559) | 0.816 |  |
| AA (continuous) | 1.0000 (0.9994, 1.0005) | 0.956 | 0.9995 (0.9988, 1.0001) | 0.103 | 0.9994 (0.9987, 1.0001) | 0.075 |  |
| Quartile 1 | ref |  | ref |  | ref |  |  |
| Quartile 2 | 1.0288 (0.7502, 1.4109) | 0.860 | 0.9462 (0.6993, 1.2801) | 0.720 | 0.8732 (0.6484, 1.1758) | 0.372 |  |
| Quartile 3 | 0.8446 (0.5976, 1.1937) | 0.339 | 0.6806 (0.4638, 0.9988) | 0.049 | 0.6325 (0.4251, 0.9409) | 0.024 |  |
| Quartile 4 | 1.0401 (0.7373, 1.4672) | 0.823 | 0.7499 (0.5140, 1.0942) | 0.135 | 0.6786 (0.4414, 1.0431) | 0.077 |  |
| DTA (continuous) | 1.0039 (0.9900, 1.0179) | 0.584 | 1.0118 (0.9956, 1.0282) | 0.154 | 1.0027 (0.9848, 1.0209) | 0.771 |  |
| Quartile 1 | ref |  | ref |  | ref |  |  |
| Quartile 2 | 1.0876 (0.7303, 1.6196) | 0.680 | 1.3529 (0.8651, 2.1157) | 0.185 | 1.1918 (0.7638, 1.8597) | 0.440 |  |
| Quartile 3 | 1.1486 (0.7470, 1.7660) | 0.528 | 1.4477 (0.8402, 2.4946) | 0.183 | 1.2216 (0.6485, 2.3011) | 0.536 |  |
| Quartile 4 | 1.1481 (0.7220, 1.8256) | 0.559 | 1.4301 (0.7746, 2.6402) | 0.253 | 1.0827 (0.5544, 2.1145) | 0.816 |  |
| n-6 DPA (continuous) | 0.9968 (0.9802, 1.0137) | 0.708 | 1.0109 (0.9911, 1.0311) | 0.282 | 1.0014 (0.9804, 1.0229) | 0.895 |  |
| Quartile 1 | ref |  | ref |  | ref |  |  |
| Quartile 2 | 0.7689 (0.5170, 1.1435) | 0.194 | 0.9125 (0.6369, 1.3074) | 0.618 | 0.8685 (0.5931, 1.2717) | 0.469 |  |
| Quartile 3 | 0.8722 (0.5582, 1.3628) | 0.548 | 1.0445 (0.6173, 1.7674) | 0.871 | 0.8653 (0.4796, 1.5609) | 0.631 |  |
| Quartile 4 | 0.9235 (0.6077, 1.4036) | 0.710 | 1.3151 (0.8196, 2.1103) | 0.256 | 1.0778 (0.6421, 1.8089) | 0.777 |  |
| Total n-3 (continuous) | | 1.0010 (1.0003, 1.0017) | 0.005 | 0.9997 (0.9990, 1.0005) | 0.508 | 1.0000 (0.9994, 1.0006) | 0.993 |
| Quartile 1 | | ref |  | ref |  | ref |  |
| Quartile 2 | | 0.8087 (0.4528, 1.4444) | 0.473 | 0.5695 (0.3297, 0.9835) | 0.043 | 0.6131 (0.3405, 1.1040) | 0.103 |
| Quartile 3 | | 0.7174 (0.4720, 1.0902) | 0.120 | 0.4122 (0.2687, 0.6326) | <0.001 | 0.4921 (0.3142, 0.7705) | 0.002 |
| Quartile 4 | | 1.1851 (0.8264, 1.6996) | 0.356 | 0.5669 (0.4085, 0.7868) | <0.001 | 0.7485 (0.5187, 1.0800) | 0.121 |
| ALA (continuous) | | 0.9995 (0.9957, 1.0034) | 0.816 | 0.9994 (0.9949, 1.0040) | 0.801 | 0.9993 (0.9947, 1.0039) | 0.756 |
| Quartile 1 | | ref |  | ref |  | ref |  |
| Quartile 2 | | 0.6526 (0.4253, 1.0015) | 0.051 | 0.5724 (0.3734, 0.8776) | 0.011 | 0.6253 (0.3960, 0.9874) | 0.044 |
| Quartile 3 | | 0.8678 (0.5661, 1.3303) | 0.515 | 0.8406 (0.5372, 1.3155) | 0.447 | 0.9063 (0.6017, 1.3651) | 0.638 |
| Quartile 4 | | 0.8564 (0.5751, 1.2752) | 0.445 | 0.7481 (0.4916, 1.1384) | 0.175 | 0.8041 (0.5040, 1.2828) | 0.360 |
| SDA (continuous) | | 1.0175 (0.9685, 1.0690) | 0.490 | 1.0078 (0.9501, 1.0690) | 0.797 | 1.0048 (0.9437, 1.0699) | 0.881 |
| Quartile 1 | | ref |  | ref |  | ref |  |
| Quartile 2 | | 1.1104 (0.5726, 2.1531) | 0.757 | 0.9838 (0.5017, 1.9293) | 0.962 | 1.0598 (0.5246, 2.1412) | 0.871 |
| Quartile 3 | | 1.4022 (0.7695, 2.5549) | 0.270 | 1.0217 (0.5426, 1.9240) | 0.947 | 0.9919 (0.5083, 1.9353) | 0.981 |
| Quartile 4 | | 1.3254 (0.7395, 2.3755) | 0.344 | 1.0663 (0.5776, 1.9683) | 0.837 | 1.1260 (0.5601, 2.2634) | 0.739 |
| EPA (continuous) | | 1.0032 (1.0019, 1.0045) | <0.001 | 1.0002 (0.9985, 1.0019) | 0.832 | 1.0009 (0.9996, 1.0022) | 0.183 |
| Quartile 1 | | ref |  | ref |  | ref |  |
| Quartile 2 | | 1.3260 (0.8176, 2.1503) | 0.253 | 0.8885 (0.5687, 1.3881) | 0.603 | 0.8825 (0.5349, 1.4559) | 0.625 |
| Quartile 3 | | 1.1479 (0.7206, 1.8284) | 0.561 | 0.6331 (0.3895, 1.0290) | 0.065 | 0.7045 (0.4349, 1.1413) | 0.155 |
| Quartile 4 | | 1.5290 (1.0448, 2.2376) | 0.029 | 0.7207 (0.5154, 1.0079) | 0.056 | 0.9179 (0.6327, 1.3317) | 0.652 |
| n-3 DPA (continuous) | | 1.0077 (1.0029, 1.0125) | 0.002 | 0.9996 (0.9934, 1.0057) | 0.887 | 0.9991 (0.9930, 1.0052) | 0.776 |
| Quartile 1 | | ref |  | ref |  | ref |  |
| Quartile 2 | | 0.7483 (0.4582, 1.2221) | 0.247 | 0.4802 (0.2962, 0.7783) | 0.003 | 0.4818 (0.3094, 0.7500) | 0.001 |
| Quartile 3 | | 1.3574 (0.9848, 1.8709) | 0.062 | 0.8568 (0.6326, 1.1604) | 0.318 | 0.8413 (0.6163, 1.1484) | 0.276 |
| Quartile 4 | | 1.4009 (0.9605, 2.0432) | 0.080 | 0.7262 (0.5128, 1.0283) | 0.071 | 0.7587 (0.4945, 1.1643) | 0.206 |
| DHA (continuous) | | 1.0021 (1.0005, 1.0037) | 0.012 | 0.9988 (0.9969, 1.0007) | 0.228 | 0.9996 (0.9983, 1.0010) | 0.606 |
| Quartile 1 | | ref |  | ref |  | ref |  |
| Quartile 2 | | 0.5374 (0.2909, 0.9928) | 0.047 | 0.3997 (0.2024, 0.7894) | 0.008 | 0.4420 (0.2338, 0.8359) | 0.012 |
| Quartile 3 | | 0.8611 (0.5971, 1.2419) | 0.423 | 0.4388 (0.3085, 0.6241) | <0.001 | 0.5316 (0.3608, 0.7833) | 0.001 |
| Quartile 4 | | 1.0802 (0.6795, 1.7173) | 0.744 | 0.4392 (0.2930, 0.6583) | <0.001 | 0.5841 (0.3878, 0.8798) | **0.010** |
| n-6/n-3 (continuous) | | 0.9155 (0.8696, 0.9638) | <0.001 | 1.0034 (0.9644, 1.0439) | 0.867 | 0.9844 (0.9450, 1.0255) | 0.451 |
| Quartile 1 | | ref |  | ref |  | ref |  |
| Quartile 2 | | 0.5995 (0.3952, 0.9094) | 0.016 | 0.7447 (0.4666, 1.1884) | 0.216 | 0.6959 (0.4489, 1.0786) | 0.105 |
| Quartile 3 | | 0.6044 (0.4288, 0.8520) | 0.004 | 1.1585 (0.8161, 1.6446) | 0.410 | 0.9317 (0.6330, 1.3712) | 0.720 |
| Quartile 4 | | 0.3309 (0.1736, 0.6310) | <0.001 | 0.8607 (0.5122, 1.4466) | 0.571 | 0.6979 (0.4104, 1.1870) | 0.184 |

Model 1: Unadjusted.

Model 2: Adjusted for age, sex, and race/ethnicity.

Model 3: Adjusted for age, sex, race/ethnicity, education, poverty income ratio, body mass index, smoking status, diabetes, hypertension, cardiovascular disease, cancer.

HR: Hazard ratio; CI: Confidence interval; ref: reference level; PhenoAgeAceel: phenotypic age acceleration.

**Supplementary Table 4. Association of serum n-6 and n-3 PUFAs with cardiovascular mortality (Weighted)**

|  | **Model 1** | | **Model 2** | | **Model 3** | |  |
| --- | --- | --- | --- | --- | --- | --- | --- |
|  | ***HR (95% CI)*** | ***p*** | ***HR (95% CI)*** | ***p*** | ***HR (95% CI)*** | ***p*** |  |
| Total n-6 (continuous) | | 0.9996 (0.9992, 0.9999) | 0.014 | 0.9997 (0.9993, 1.0000) | 0.059 | 0.9997 (0.9994, 1.0000) | 0.040 |
| Quartile 1 | | Ref |  | Ref |  | Ref |  |
| Quartile 2 | | 0.5031 (0.2853, 0.8873) | 0.018 | 0.6781 (0.3973, 1.1575) | 0.155 | 0.7609 (0.4476, 1.2934) | 0.313 |
| Quartile 3 | | 0.3336 (0.1884, 0.5907) | <0.001 | 0.4080 (0.1900, 0.8760) | 0.021 | 0.4648 (0.2365, 0.9136) | 0.026 |
| Quartile 4 | | 0.3532 (0.1748, 0.7133) | 0.004 | 0.4546 (0.2086, 0.9907) | 0.047 | 0.4636 (0.2249, 0.9554) | 0.037 |
| LA (continuous) | | 0.9994 (0.9989, 0.9998) | 0.008 | 0.9996 (0.9992, 1.0000) | 0.036 | 0.9996 (0.9994, 0.9999) | 0.017 |
| Quartile 1 | | Ref |  | Ref |  | Ref |  |
| Quartile 2 | | 0.3718 (0.1916, 0.7215) | 0.003 | 0.5774 (0.3090, 1.0789) | 0.085 | 0.6762 (0.3496, 1.3080) | 0.245 |
| Quartile 3 | | 0.2669 (0.1263, 0.5637) | <0.001 | 0.4832 (0.2135, 1.0938) | 0.081 | 0.5606 (0.2555, 1.2299) | 0.149 |
| Quartile 4 | | 0.2718 (0.1389, 0.5318) | <0.001 | 0.3701 (0.1918, 0.7142) | 0.003 | 0.3924 (0.2272, 0.6779) | <0.001 |
| GLA (continuous) | | 0.9977 (0.9879, 1.0076) | 0.648 | 1.0012 (0.9883, 1.0143) | 0.854 | 1.0010 (0.9886, 1.0135) | 0.880 |
| Quartile 1 | | Ref |  | Ref |  | Ref |  |
| Quartile 2 | | 1.2059 (0.5161, 2.8175) | 0.666 | 1.0315 (0.4056, 2.6236) | 0.948 | 1.1181 (0.4083, 3.0618) | 0.828 |
| Quartile 3 | | 1.0640 (0.5073, 2.2319) | 0.870 | 1.0459 (0.4365, 2.5060) | 0.920 | 0.9427 (0.3853, 2.3065) | 0.897 |
| Quartile 4 | | 0.9895 (0.3566, 2.7453) | 0.984 | 1.1841 (0.3123, 4.4895) | 0.804 | 1.3023 (0.3173, 5.3451) | 0.714 |
| EDA (continuous) | | 0.9766 (0.9285, 1.0271) | 0.357 | 0.9698 (0.9082, 1.0355) | 0.359 | 0.9623 (0.9113, 1.0162) | 0.167 |
| Quartile 1 | | Ref |  | Ref |  | Ref |  |
| Quartile 2 | | 0.7276 (0.3379, 1.5671) | 0.417 | 0.5698 (0.2788, 1.1642) | 0.123 | 0.5795 (0.2603, 1.2900) | 0.181 |
| Quartile 3 | | 0.5031 (0.2144, 1.1804) | 0.114 | 0.3820 (0.1750, 0.8337) | 0.016 | 0.3933 (0.1795, 0.8619) | 0.020 |
| Quartile 4 | | 0.5031 (0.2362, 1.0718) | 0.075 | 0.4279 (0.2119, 0.8641) | 0.018 | 0.3602 (0.1791, 0.7245) | 0.004 |
| HGLA (continuous) | | 0.9950 (0.9897, 1.0003) | 0.063 | 0.9983 (0.9919, 1.0047) | 0.605 | 0.9986 (0.9924, 1.0049) | 0.662 |
| Quartile 1 | | Ref |  | Ref |  | Ref |  |
| Quartile 2 | | 0.6305 (0.3407, 1.1667) | 0.142 | 0.6226 (0.3356, 1.1548) | 0.133 | 0.5815 (0.3115, 1.0857) | 0.089 |
| Quartile 3 | | 0.5925 (0.2816, 1.2467) | 0.168 | 0.9075 (0.3894, 2.1152) | 0.822 | 1.0413 (0.4485, 2.4176) | 0.925 |
| Quartile 4 | | 0.3155 (0.0975, 1.0206) | 0.054 | 0.5050 (0.1298, 1.9644) | 0.324 | 0.4794 (0.1258, 1.8273) | 0.282 |
| AA (continuous) | | 0.9999 (0.9989, 1.0010) | 0.888 | 0.9996 (0.9983, 1.0009) | 0.537 | 0.9995 (0.9979, 1.0010) | 0.490 |
| Quartile 1 | | Ref |  | Ref |  | Ref |  |
| Quartile 2 | | 0.6999 (0.3388, 1.4459) | 0.335 | 0.6457 (0.3399, 1.2268) | 0.182 | 0.5315 (0.2620, 1.0781) | 0.080 |
| Quartile 3 | | 0.7421 (0.4596, 1.1981) | 0.222 | 0.5913 (0.3173, 1.1020) | 0.098 | 0.4994 (0.2614, 0.9542) | 0.036 |
| Quartile 4 | | 0.7990 (0.3998, 1.5966) | 0.525 | 0.6623 (0.2986, 1.4691) | 0.311 | 0.5539 (0.2246, 1.3657) | 0.199 |
| DTA (continuous) | | 0.9953 (0.9648, 1.0267) | 0.765 | 1.0140 (0.9802, 1.0490) | 0.420 | 1.0073 (0.9729, 1.0430) | 0.680 |
| Quartile 1 | | Ref |  | Ref |  | Ref |  |
| Quartile 2 | | 1.4150 (0.6968, 2.8733) | 0.337 | 1.9567 (0.8872, 4.3154) | 0.096 | 1.7501 (0.8387, 3.6520) | 0.136 |
| Quartile 3 | | 0.6200 (0.2051, 1.8737) | 0.397 | 0.8944 (0.2918, 2.7408) | 0.845 | 0.7662 (0.3073, 1.9106) | 0.568 |
| Quartile 4 | | 0.8365 (0.2527, 2.7688) | 0.770 | 1.4228 (0.4004, 5.0565) | 0.586 | 1.0586 (0.2884, 3.8855) | 0.932 |
| n-6 DPA (continuous) | | 0.9923 (0.9625, 1.0230) | 0.620 | 1.0172 (0.9881, 1.0472) | 0.249 | 1.0109 (0.9796, 1.0432) | 0.499 |
| Quartile 1 | | Ref |  | Ref |  | Ref |  |
| Quartile 2 | | 0.3393 (0.1577, 0.7296) | 0.006 | 0.4146 (0.1863, 0.9229) | 0.031 | 0.4343 (0.2071, 0.9106) | 0.027 |
| Quartile 3 | | 0.5104 (0.2267, 1.1492) | 0.104 | 0.7343 (0.3319, 1.6246) | 0.446 | 0.6796 (0.3101, 1.4893) | 0.335 |
| Quartile 4 | | 0.7875 (0.4087, 1.5174) | 0.475 | 1.4774 (0.7094, 3.0770) | 0.297 | 1.2366 (0.5566, 2.7470) | 0.602 |
| Total n-3 (continuous) | | 1.0007 (0.9991, 1.0022) | 0.398 | 0.9991 (0.9968, 1.0015) | 0.463 | 0.9990 (0.9971, 1.0009) | 0.321 |
| Quartile 1 | | Ref |  | Ref |  | Ref |  |
| Quartile 2 | | 0.7348 (0.3967, 1.3609) | 0.327 | 0.4972 (0.2455, 1.0069) | 0.052 | 0.5294 (0.2115, 1.3254) | 0.174 |
| Quartile 3 | | 0.7550 (0.3953, 1.4421) | 0.395 | 0.3864 (0.1968, 0.7587) | 0.006 | 0.3730 (0.1795, 0.7750) | 0.008 |
| Quartile 4 | | 1.3069 (0.6551, 2.6071) | 0.447 | 0.5642 (0.2698, 1.1800) | 0.128 | 0.6059 (0.3154, 1.1637) | 0.132 |
| ALA (continuous) | | 0.9979 (0.9870, 1.0088) | 0.700 | 0.9980 (0.9879, 1.0083) | 0.706 | 0.9967 (0.9874, 1.0061) | 0.495 |
| Quartile 1 | | Ref |  | Ref |  | Ref |  |
| Quartile 2 | | 0.8270 (0.4423, 1.5464) | 0.552 | 0.7074 (0.3884, 1.2884) | 0.258 | 0.7061 (0.4042, 1.2335) | 0.221 |
| Quartile 3 | | 0.5646 (0.2734, 1.1663) | 0.123 | 0.6355 (0.3125, 1.2921) | 0.210 | 0.5804 (0.2920, 1.1539) | 0.121 |
| Quartile 4 | | 0.7865 (0.3502, 1.7663) | 0.561 | 0.7069 (0.3461, 1.4439) | 0.341 | 0.6774 (0.3474, 1.3209) | 0.253 |
| SDA (continuous) | | 1.0007 (0.8608, 1.1634) | 0.992 | 1.0174 (0.8630, 1.1993) | 0.837 | 0.9916 (0.8205, 1.1985) | 0.931 |
| Quartile 1 | | Ref |  | Ref |  | Ref |  |
| Quartile 2 | | 1.1550 (0.6196, 2.1529) | 0.650 | 1.0855 (0.5649, 2.0858) | 0.806 | 1.3696 (0.7099, 2.6424) | 0.348 |
| Quartile 3 | | 1.3470 (0.7085, 2.5611) | 0.364 | 1.0381 (0.4860, 2.2172) | 0.923 | 0.9261 (0.4500, 1.9057) | 0.835 |
| Quartile 4 | | 1.0963 (0.4097, 2.9337) | 0.855 | 1.0212 (0.4141, 2.5185) | 0.964 | 0.9570 (0.4060, 2.2563) | 0.920 |
| EPA (continuous) | | 1.0029 (1.0009, 1.0049) | 0.004 | 0.9997 (0.9956, 1.0038) | 0.869 | 0.9995 (0.9961, 1.0029) | 0.768 |
| Quartile 1 | | Ref |  | Ref |  | Ref |  |
| Quartile 2 | | 1.0623 (0.3325, 3.3941) | 0.919 | 0.6838 (0.2063, 2.2667) | 0.534 | 0.6585 (0.2135, 2.0312) | 0.467 |
| Quartile 3 | | 1.7945 (0.7163, 4.4957) | 0.212 | 0.9184 (0.3336, 2.5287) | 0.869 | 0.9416 (0.3073, 2.8851) | 0.916 |
| Quartile 4 | | 2.1150 (0.8602, 5.2000) | 0.103 | 1.0018 (0.3795, 2.6443) | 0.997 | 1.0518 (0.3969, 2.7874) | 0.919 |
| n-3 DPA (continuous) | | 1.0066 (0.9962, 1.0172) | 0.215 | 0.9988 (0.9874, 1.0103) | 0.840 | 0.9961 (0.9860, 1.0062) | 0.446 |
| Quartile 1 | | Ref |  | Ref |  | Ref |  |
| Quartile 2 | | 0.6771 (0.2612, 1.7552) | 0.422 | 0.3943 (0.1437, 1.0814) | 0.071 | 0.3770 (0.1381, 1.0290) | 0.057 |
| Quartile 3 | | 1.0008 (0.4582, 2.1861) | 0.998 | 0.6613 (0.3191, 1.3706) | 0.266 | 0.6006 (0.2735, 1.3192) | 0.204 |
| Quartile 4 | | 1.2044 (0.6091, 2.3816) | 0.593 | 0.6246 (0.3320, 1.1751) | 0.144 | 0.5658 (0.2646, 1.2102) | 0.142 |
| DHA (continuous) | | 1.0015 (0.9986, 1.0044) | 0.318 | 0.9968 (0.9914, 1.0022) | 0.239 | 0.9967 (0.9920, 1.0013) | 0.163 |
| Quartile 1 | | Ref |  | Ref |  | Ref |  |
| Quartile 2 | | 0.7897 (0.3434, 1.8162) | 0.579 | 0.5021 (0.2286, 1.1025) | 0.086 | 0.5679 (0.2290, 1.4085) | 0.222 |
| Quartile 3 | | 0.9620 (0.3682, 2.5130) | 0.937 | 0.3723 (0.1549, 0.8947) | 0.027 | 0.4488 (0.1750, 1.1513) | 0.096 |
| Quartile 4 | | 1.6094 (0.8541, 3.0327) | 0.141 | 0.5010 (0.2533, 0.9908) | 0.047 | 0.5530 (0.3023, 1.0118) | 0.055 |
| n-6/n-3 (continuous) | | 0.8729 (0.7953, 0.9581) | 0.004 | 0.9868 (0.8838, 1.1018) | 0.813 | 0.9934 (0.9020, 1.0941) | 0.893 |
| Quartile 1 | | Ref |  | Ref |  | Ref |  |
| Quartile 2 | | 0.9036 (0.4611, 1.7710) | 0.768 | 1.1709 (0.6273, 2.1856) | 0.620 | 1.3245 (0.6826, 2.5701) | 0.406 |
| Quartile 3 | | 0.3606 (0.1424, 0.9132) | 0.031 | 0.8275 (0.3022, 2.2661) | 0.713 | 0.8311 (0.3271, 2.1113) | 0.697 |
| Quartile 4 | | 0.2283 (0.0948, 0.5499) | <0.001 | 0.8844 (0.3072, 2.5463) | 0.820 | 0.9172 (0.3463, 2.4293) | 0.862 |

Model 1: Unadjusted.

Model 2: Adjusted for age, sex, and race/ethnicity.

Model 3: Adjusted for age, sex, race/ethnicity, education, poverty income ratio, body mass index, smoking status, diabetes, hypertension, cardiovascular disease, cancer.

HR: Hazard ratio; CI: Confidence interval; ref: reference level; PhenoAgeAceel: phenotypic age acceleration.

1. **Supplementaryary Methods**

**Supplementary method 1.** Assessment of biological age

The PhenoAge algorithm was derived using an elastic-net penalized Gompertz regression model, with mortality as the outcome variable. Levine et al. applied a Cox proportional hazards model with elastic-net regularization and 10-fold cross-validation to select the most informative biomarkers from an initial set of 42 in NHANES III. The final model included chronological age and nine biomarkers, with beta coefficients determined through the penalized Cox regression approach. The study identified eight key biomarkers, along with chronological age, to calculate PhenoAge, including albumin, alkaline phosphatase, creatinine, glycated hemoglobin, white blood cell count, lymphocyte percentage, mean cell volume, and red cell distribution width. The detail formula is as follows:

$$xb=-18.312-0.0292\times albumin+0.0023\times alkaline phosphatase+0.0064\times creatinine+0.1778\times glycated hemoglobin+0.0552\times white blood cell count-0.0135\times lymphocyte percentage+0.0293\times mean corpuscular volume+0.2345\times red cell distribution width+0.0772\times chronological age$$

$$\gamma=0.007354285$$

$$mortality risk=1-e^{{-e}^{\frac{{xb}_{{[exp(120}_{x\gamma})-1]}}{\gamma}}}$$

$$PhenoAge=143.5671+\frac{\ln\left[ -0.0059383581 \times\ln\left( 1-mortality risk \right) \right]}{0.08548908}$$
